# Confidence Intervals and Sample Size for the ICC in Two‐Way ANOVA Models

**DOI:** 10.1002/sim.70106

**Published:** 2025-05-22

**Authors:** Dipro Mondal, Math J J M Candel, Alberto Cassese, Sophie Vanbelle

**Affiliations:** ^1^ Department of Methodology and Statistics Care and Public Health Research Institute (CAPHRI), Maastricht University Maastricht the Netherlands; ^2^ Department of Statistics Computer Science, Applications “Giuseppe Parenti” The University of Florence Firenze Italy

**Keywords:** interrater reliability, intrarater reliability, measurement errors, observer variation, reproducibility of results

## Abstract

The reliability of measurement instruments is vital in fields like medicine and psychology, where these tools are often used for diagnostic purposes. In reliability studies where participants are assessed by the same set of raters, the data can be modeled using a two‐way ANOVA, with the intraclass correlation coefficient (ICC) as a key metric. This paper focuses on the ICC for agreement, which is crucial when the measurement values themselves, rather than just their rank ordering, are of interest. However, selecting appropriate confidence interval methods and determining adequate sample sizes for the ICC for agreement remains challenging. This work advances the understanding of confidence interval methods for the ICC for agreement, provides practical tools, and offers recommendations for selecting confidence interval methods and sample size procedures for planning reliability studies. In particular, we provide a comprehensive review and simulation‐based comparison of six classes of confidence interval methods for the ICC for agreement identified in the literature. Our analysis includes a method based on the F‐distribution, previously omitted, which demonstrates the best statistical properties in some cases. Then, in conjunction with the best‐performing methods, we evaluate three sample size determination procedures based on the expected width of confidence intervals that we identified in the literature. To address the lack of accessible tools, we further developed an interactive R Shiny app, freely available to researchers, to compute confidence intervals and sample sizes. The utility of these methods is illustrated by a study on fetal heart rates.

## 1 Introduction

The reliability of measurement instruments plays an important role in numerous scientific disciplines, such as medicine and psychology, where these instruments, amongst others, are used for diagnostic purposes. A reliability study is generally performed to assess the reliability of these instruments (i.e., how well the instrument is able to distinguish among participants in a population), which comprises participants measured repeatedly under similar conditions by the same instrument or rater (intrarater reliability), or by different instruments or raters (interrater reliability).

A widely used metric to evaluate reliability when the measurements are quantitative is the intraclass correlation coefficient (ICC), which can take different forms depending on the study design [1, 2]. This paper focuses on the ICC for a reliability study where some participants are measured by the same set of raters. A suitable model for this data is the two‐way analysis of variance (ANOVA) model. When different sets of raters rate each participant, or when repeated measurements are made simultaneously on each participant by the same rater, and these measurements for a participant are exchangeable, a one‐way ANOVA model can be used. For a review in this context, see Mondal et al. [3]. Under a two‐way ANOVA model, two different ICC coefficients can be defined based on whether the systematic differences between the instruments/raters are relevant or not: The ICC for agreement and the ICC for consistency, respectively. The ICC for consistency is concerned with the rank ordering of the participants. In that case, whether a particular rater systematically assigns higher or lower scores relative to the other raters is irrelevant, as only the ranking is important [1]. The ICC for agreement, on the other hand, is concerned with the actual values of the measurements and takes into account the systematic differences between the raters as well [1]. In disciplines such as medicine, where measurements are used for diagnosing participants, the focus extends beyond merely rank ordering individuals to the actual values of the measurements. In such cases, the intraclass correlation coefficient (ICC) for agreement becomes crucial, which is the primary focus of this paper.

When planning a reliability study, researchers often face the challenge of determining an adequate sample size for the study. The procedures for determining the minimum number of participants and raters for the reliability study are based on the expected width of the confidence interval around a planned value for the ICC. In this procedure, the aim is to determine the minimum number of raters/participants (or a combination thereof) such that the expected width of the confidence interval is less than a pre‐specified value. Therefore, the procedure relies on a confidence interval for the ICC.

A search through existing literature on the confidence interval for the ICC for agreement reveals mainly six different classes of confidence interval methods to obtain a confidence interval, namely the moment approximation method [4, 5], the modified profile likelihood method [6], the delta method [7, 8], the modified large sample method [9, 10, 11], the generalized confidence interval method [12] and the variance partitioning method [13]. Most of these methods also include some variations in how they were developed by different authors. Comparative studies indicated that the two‐moment approximation method exhibited worse statistical properties [14, 15], while the generalized confidence interval showed better statistical properties compared to the others [11, 16]. However, these results cannot be considered final, as no study has simultaneously compared all the available confidence interval methods. For instance, methods based on F‐ and beta‐distributions [13] have been proposed but excluded from prior comparative studies. Additionally, differences in simulation settings across studies pose challenges in drawing definitive conclusions. These gaps underscore the need for a comprehensive evaluation of all available methods for constructing confidence intervals for the ICC for agreement.

Several approaches to determine the minimum number of raters and/or participants were developed to obtain a certain expected width of the confidence interval. These procedures differ based on whether the number of participants or raters is fixed [7, 17, 18]. However, their practical application is hindered by the complexity of implementation and the unavailability of user‐friendly software. These challenges frequently require advanced proficiency in programming and statistical methods, posing significant obstacles for practical use.

Our contribution directly addresses these gaps. First, we review the six classes of confidence interval methods available in the literature and complete their comparison, based on their statistical properties under some realistic scenarios, by means of a simulation study. Specifically, we make a comprehensive comparison of the available confidence interval methods, as well as those previously overlooked, and evaluate their performance in terms of their coverage and average confidence interval width. Second, for the methods with the best coverage properties, we review the different procedures to obtain a sample size for a reliability study under the two‐way ANOVA model. Third, we develop an R‐Shiny application capable of providing end‐users with an interactive way of obtaining sample sizes, using the methods described in the paper. Our work not only advances the understanding of confidence interval methods, but also equips researchers with practical tools and guidance for choosing an appropriate confidence interval method and a sample size determination procedure to plan a reliability study.

The review is organized as follows. Section 2 introduces the ICC for agreement and consistency in the context of a reliability study where each of a group of raters assesses participants once, and the data is modeled using a two‐way ANOVA model. Section 3 provides the different methods to construct a confidence interval for the ICC for agreement. Section 4 describes a Monte‐Carlo simulation setup to evaluate the different confidence interval methods and presents the result of the comparisons. Section 5 provides different sample size determination procedures. Section 6 provides an example of how the sample size determination procedures can be used in a reliability study. Finally, Section 7 provides a discussion on the various methods and procedures described in this review.

## 2 Definition and Estimation of the Intraclass Correlation Coefficients

Consider a study in which each of n participants is measured once by the same k raters. If these measurements are quantitative, they can be modeled by a two‐way ANOVA model. The outcome $y_{ij}$ for participant i (i=1,2,…,n) and rater j (j=1,2,…,k), $y_{ij}$, can then be written as [1],

(1) $$y_{ij}=\mu +s_{i}+r_{j}+e_{ij},$$

where μ is the grand mean, $s_{i}$ is the participant random effect ($s_{i}∼𝒩(0,\sigma_{s}^{2})$), $r_{j}$ is the rater effect, and $e_{ij}$ is the error ($e_{ij}∼𝒩(0,\sigma_{e}^{2})$). The rater effect can be considered as random or fixed. With the intention of generalizing the reliability to any set of raters from the rater population, we consider the rater effects as random ($r_{j}∼𝒩(0,\sigma_{r}^{2})$) as it is customary in inter‐rater reliability studies. Note that in Equation (1), no interaction term is included as it is not possible to estimate the interaction effect between participants and raters when there is only one observation for each participant‐rater combination.

Table 1 gives the variance decomposition of the ANOVA model with the mean squares denoted by MSS ( $=\frac{k}{n-1}\sum_{i=1}^{n}{\left({\bar{y}}_{i.}-{\bar{y}}_{..}\right)}^{2}$ ), MSR ( $=\frac{n}{k-1}\sum_{j=1}^{k}{\left({\bar{y}}_{.j}-\bar{y}..\right)}^{2}$ ) and MSE ( $=\frac{1}{\left(n-1)(k-1\right)}\sum_{i=1}^{n}\sum_{j=1}^{k}{\left(y_{ij}-{\bar{y}}_{i.}-{\bar{y}}_{.j}+{\bar{y}}_{..}\right)}^{2}$ ), where ${\bar{y}}_{i.}=\frac{1}{k}\sum_{j=1}^{k}y_{ij}$, ${\bar{y}}_{.j}=\frac{1}{n}\sum_{i=1}^{n}y_{ij}$ and ${\bar{y}}_{..}=\frac{1}{nk}\sum_{i=1}^{n}\sum_{j=1}^{k}y_{ij}$. The assumptions of the ANOVA model we have adopted imply that the outcomes are conditionally normal, and the mean squares follow independent scaled $\chi^{2}$‐distributions. The $\chi^{2}$‐distributions corresponding to the mean squares are given in Section 3.1.1. Using the variance decomposition, the ICC for agreement for the two‐way ANOVA model Equation (1) is defined as,

(2) $$\rho_{a}=\frac{\sigma_{s}^{2}}{\sigma_{s}^{2}+\sigma_{r}^{2}+\sigma_{e}^{2}},\;0\leq \rho_{a}\leq 1$$

and the ICC for consistency for the same model is defined as,

(3) $$\rho_{c}=\frac{\sigma_{s}^{2}}{\sigma_{s}^{2}+\sigma_{e}^{2}},\;\;0\leq \rho_{c}\leq 1.$$

The coefficient $\rho_{a}$ is concerned with absolute measurements rather than just the rank ordering of the participants as given by $\rho_{c}$. The difference between the two coefficients, $\rho_{a}$ and $\rho_{c}$, lies in the denominator. Specifically, for $\rho_{a}$ the denominator includes the systematic differences between the raters, as expressed by $\sigma_{r}^{2}$, thereby accounting for the variation from all sources (see Equation 1), unlike $\rho_{c}$. As a consequence, $\rho_{a}$ yields lower values than $\rho_{c}$.

**TABLE 1:** Variance decomposition for the ANOVA model Equation (1).

Sources of Variation	Degrees of Freedom	Mean Squares	Expected Mean Squares
Between participants	n−1	MSS	$k\sigma_{s}^{2}+\sigma_{e}^{2}$
Between raters	k−1	MSR	$n\sigma_{r}^{2}+\sigma_{e}^{2}$
Errors	(n−1)(k−1)	MSE	$\sigma_{e}^{2}$

Under the ANOVA model given by Equation (1), the aforementioned ICC for agreement is estimated as [1, 19],

(4) $${\hat{\rho }}_{a}=\frac{MSS-MSE}{MSS+(k-1)MSE+\frac{k}{n}(MSR-MSE)}$$

and that for consistency is estimated as [1],

(5) $${\hat{\rho }}_{c}=\frac{MSS-MSE}{MSS+(k-1)MSE}.$$

Note that Almehrizi and Emam [8] provided an alternative expression for ${\hat{\rho }}_{a}$ and for ${\hat{\rho }}_{c}$ using a matrix formulation. The expression for $\rho_{a}$ is provided in Equation (8). Readers interested in the estimation of ICC for other cases, such as when the rater effects are considered fixed, can consult McGraw and Wong [1]. In the following sections, we focus only on the ICC for agreement. Henceforth we denote $\rho_{a}=\rho$ and its estimator by ${\hat{\rho }}_{a}=\hat{\rho }$.

## 3 Confidence Intervals for the ICC for Agreement

In the literature, there are six classes of confidence interval methods to construct the lower (L) and upper (U) limits of the confidence interval for ρ. These are the moment approximation method [4, 5], the modified profile likelihood method [6], the delta method [7, 8], the modified large sample method [9, 10, 11], the generalized confidence interval method [12], and the variance partitioning method [13]. In this study, we do not evaluate the moment approximation and modified profile likelihood methods and focus on the latter four methods. The details of these two methods are provided in Supporting Information Data S1 (Section A), and the rationale for their exclusion is discussed in Section 4. Below, we summarize the most important features of the four classes of confidence interval methods we will evaluate.

### 3.1 Delta Method

The delta method allows us to derive analytical approximations for the variance of $\hat{\rho }$ (or a transformation of it). This method was used by Saito et al. [7] to derive an approximation for the asymptotic variance of the log‐transformed $\hat{\rho }$, $V(log(\hat{\rho }))$, and by Almehrizi and Emam [8] to derive an expression for the approximate variance of $\hat{\rho }$ using matrix algebra.

#### 3.1.1 Delta Method Applied to Log‐Transformation ($W_{log}$)

Saito et al. [7] derived an approximate expression of the asymptotic variance, $V(\hat{\rho })$, based on the delta method. The variance on the log‐transformed scale, $log(\hat{\rho })$ is,

(6) $$\begin{array}{ll} & V(log(\hat{\rho }))\\ & \;=\frac{V(MSS)+V(MSE)}{{\left[E\left(MSS\right)‐E\left(MSE\right)\right]}^{2}}\\ & \;+\frac{V(MSS)+{\left(k/n\right)}^{2}V(MSR)+{\left(k-1-k/n\right)}^{2}V(MSE)}{{\left[E(MSS)+(k/n)E(MSR)+(k-1-k/n)E(MSE)\right]}^{2}}\\ & \;-2\frac{V(MSS)-(k-1-k/n)V(MSE)}{\begin{array}{c} \left[E(MSS)-E(MSE)][E(MSS)+(k/n)E(MSR)+(k-1-k/n)E(MSE)\right] \end{array}}. \end{array}$$

Since following from the assumptions of the ANOVA model in Equation (1), $MSS∼\chi_{n-1}^{2}\frac{k\sigma_{s}^{2}+\sigma_{e}^{2}}{n-1}$, $MSR∼\chi_{k-1}^{2}\frac{n\sigma_{r}^{2}+\sigma_{e}^{2}}{k-1}$ and $MSE∼\chi_{\left(n-1)(k-1\right)}^{2}\frac{\sigma_{e}^{2}}{\left(k-1)(n-1\right)}$ where $\chi_{n-1}^{2}$, $\chi_{k-1}^{2}$ and $\chi_{\left(n-1)(k-1\right)}^{2}$ are a set of jointly independent $\chi^{2}$ random variables, their expected values and variances can be obtained as,

$$\begin{array}{ll} & E(MSS)=k\sigma_{s}^{2}+\sigma_{e}^{2},\;\;V(MSS)=\frac{2{\left(k\sigma_{s}^{2}+\sigma_{e}^{2}\right)}^{2}}{n-1},\\ & E(MSR)=n\sigma_{r}^{2}+\sigma_{e}^{2},\;\;V(MSR)=\frac{2{\left(n\sigma_{r}^{2}+\sigma_{e}^{2}\right)}^{2}}{k-1},\\ & E(MSE)=\sigma_{e}^{2},\;\;V(MSE)=\frac{2\sigma_{e}^{4}}{\left(k-1)(n-1\right)}. \end{array}$$

Substituting these into Equation (6) we obtain the final expression for $V(log(\hat{\rho }))$. The estimate of $V(log(\hat{\rho }))$, $\hat{V}(log(\hat{\rho }))$ is obtained by plugging in the estimates of $\sigma_{s}^{2}$, $\sigma_{r}^{2}$ and $\sigma_{e}^{2}$ in Equation (6). The (1−α)×100% confidence interval for log(ρ) can be constructed using the Wald confidence interval method as,

(7) $$L_{log},U_{log}=log(\hat{\rho })\pm Z(1-\alpha /2)\sqrt{\hat{V}(log(\hat{\rho }))},$$

where the expression of $\hat{V}(log(\hat{\rho }))$ is computed as in Equation (6), and $Z(1-\frac{\alpha }{2})$ denotes the $(1-\frac{\alpha }{2})\times 100$ percentile of a standard normal distribution. The confidence interval for ρ is obtained by exponentiating $L_{log}$ and $U_{log}$.

#### 3.1.2 Delta Method Applied Directly With Matrix Formulation ($W_{mat}$)

Almehrizi and Emam [8] obtained the expression for $\hat{\rho }$ Equation (4) in matrix formulation as,

(8) $$\hat{\rho }=\frac{1^{\prime }S1-trS}{\frac{1}{n}1^{\prime }S1+\frac{nk-n-k}{n}trS+ky_{d}^{\prime }y_{d}},$$

where 1 is a row vector of 1's (k×1), S is the estimated sample variance‐covariance matrix of k measurements (k×k), and $y_{d}={\left({\bar{y}}_{.1}-{\bar{y}}_{..},\ldots ,{\bar{y}}_{.k}-{\bar{y}}_{..}\right)}^{\prime }$ is a (k×1) vector of deviations of average measurements of each rater j (${\bar{y}}_{.j}$) from the grand average (${\bar{y}}_{..}$). Note that, $1^{\prime }S1=kMSS=\frac{k^{2}}{n-1}\sum_{i=1}^{n}{\left({\bar{y}}_{i.}-{\bar{y}}_{..}\right)}^{2}$ is the sample variance of total measurements of participants, $trS=MSS+(k-1)MSE=\sum_{j=1}^{k}(\frac{1}{n-1}\sum_{i=1}^{n}{\left(y_{ij}-{\bar{y}}_{.j}\right)}^{2})$ is the sum of within rater sample variances, and, $y_{d}^{\prime }y_{d}=\frac{k-1}{n}MSR=\sum_{j=1}^{k}{\left({\bar{y}}_{.j}-{\bar{y}}_{..}\right)}^{2}$ is the sum of squared deviations of average measurements of each rater j from the grand average. The three main terms $1^{\prime }S1$, trS, and $y_{d}^{\prime }y_{d}$ are non‐linear functions of the elements of the data matrix. Therefore, by the delta method, the estimated variance‐covariance matrix of the three terms $\frac{n-1}{n}1^{\prime }\;S1$, $\frac{n-1}{n}trS$, and $y_{d}^{\prime }y_{d}$ for the given model Equation (1) can be obtained as [8],

$${\hat{V}}_{y}=\left(\begin{array}{ccc} \frac{2{\left(1^{\prime }S1\right)}^{2}}{n+1} & \frac{2\left(1^{\prime }S^{2}1\right)}{n+1} & 0\\ \frac{2\left(1^{\prime }S^{2}1\right)}{n+1} & \frac{2\left(trS^{2}\right)}{n+1} & 0\\ 0 & 0 & \frac{2{\left(y_{d}^{\prime }y_{d}\right)}^{2}}{k+1} \end{array}\right).$$

Then the estimated variance of the estimator of ρ is,

(9) $$\begin{array}{ll} & \hat{V}(\hat{\rho })\\ & \;=\frac{2{\hat{\rho }}^{2}\left[{\left(1+\frac{nk-n-k}{n}\hat{\rho }\right)}^{2}trS^{2}+{\left(1-\frac{\hat{\rho }}{n}\right)}^{2}{\left(1^{\prime }S1\right)}^{2}\left.-2\left(1+\frac{nk-n-k}{n}\hat{\rho }\right)\left(1-\frac{\hat{\rho }}{n}\right)1^{\prime }S^{2}1\right]\right.}{(n+1){\left[1^{\prime }S1-trS\right]}^{2}}\\ & \;+\frac{8k{\hat{\rho }}^{4}y_{d}^{\prime }y_{d}}{{\left[1^{\prime }S1-trS\right]}^{2}} \end{array}$$

The confidence interval for ρ is then obtained as [8],

$$\begin{array}{l} L,U=\hat{\rho }\pm T_{n+1}(1-\frac{\alpha }{2})\sqrt{\hat{V}(\hat{\rho })}, \end{array}$$

where $\hat{V}(\hat{\rho })$ is replaced by Equation (9), and $T_{n+1}(1-\frac{\alpha }{2})$ is the $(1-\frac{\alpha }{2})\times 100$ percentile of the t‐distributionwith n+1 degrees of freedom. Note that Almehrizi and Emam [8] use a t‐distribution instead of the generally used normal distribution.

### 3.2 Modified Large Sample Method

The modified large sample method introduced by Graybill and Wang [20] aims to find a refined approximation of the confidence interval for ρ starting with an approximate expression of a large sample confidence interval involving mean squares. The initial approximation is improved under limiting conditions of each of the variance components involved, thereby obtaining a modified large sample approximation of the confidence interval. Note that the final expression of the confidence limits may be different depending on the conditions for exactness that were defined during its derivation (see Burdick et al. [21] for further details).

Arteaga et al. [9] and Gui et al. [10] derived the confidence interval for ρ using the modified large sample method, each starting from different assumptions. The corresponding two‐sided confidence intervals are different. Note that Shoukri [22] incorrectly refers to the hybrid moment method described in Supporting Information Data S1 (Section A.1.2) as the modified large sample method.

#### 3.2.1 Modified Large Sample Confidence Interval for ρ by Arteaga Et Al. [9]($MLS_{A}$)

Arteaga et al. [9] derived the confidence limits for the transformation $\frac{k\rho }{n(1-\rho )}$ as,

(10)  LA∗=−1+Fn−1,∞−1(1−α2)F1+(1−Fn−1,∞−1(1−α2)F5)F5F1−1n−1+Fn−1,∞−1(1−α2)F3F2 and UA∗=−1+Fn−1,∞−1(α2)F1+(1−Fn−1,∞−1(α2)F6)F6F1−1n−1+Fn−1,∞−1(α2)F4F2

where $F_{1}=\frac{MSS}{MSE}$, $F_{2}=\frac{MSR}{MSE}$, $F_{3}=F_{n-1,k-1}(1-\frac{\alpha }{2})$, $F_{4}=F_{n-1,k-1}(\frac{\alpha }{2})$ $F_{5}=F_{n-1,(n-1)(k-1)}(1-\frac{\alpha }{2})$ and $F_{6}=F_{n-1,(n-1)(k-1)}\times (\frac{\alpha }{2})$. Then, the limits of the confidence interval for ρ are,

$$\begin{array}{l} L=\frac{n\;\max(0,L_{A}^{*})}{k+n\;\max(0,L_{A}^{*})},\;\text{and}\;U=\frac{n\;\max(0,U_{A}^{*})}{k+n\;\max(0,U_{A}^{*})}, \end{array}$$

where $L_{A}^{*}$ and $U_{A}^{*}$ are given in Equation (10). Henceforth, we refer to this method as $MLS_{A}$. Note that the expressions of $L_{A}^{*}$ and $U_{A}^{*}$ in Equation 2.9 from Arteaga et al. [9] is incorrect (see Appendix C for the correction of the formula in the paper of Arteaga et al. [9]).

#### 3.2.2 Modified Large Sample Confidence Interval for ρ by Gui Et Al. [10] ($MLS_{G}$)

Gui et al. [10] proposed an expression of the confidence interval according to the modified large sample method based on a general form of a correlation coefficient which was adapted by Cappelleri and Ting [15] for ρ. The final expressions of the limits of the confidence interval are quite elaborate, so we refer to Appendix A of Cappelleri and Ting [15] for the exact expressions. Henceforth, we refer to this method as $MLS_{G}$. Note that, the expression of the limits of the confidence interval presented in Appendix B of Ionan et al. [11] corresponds to expressions for $MLS_{A}$, although it is referred to as the method by Cappelleri and Ting [15]. However, their evaluation of the accuracy of the confidence interval methods appears to use the correct expression for $MLS_{G}$.

### 3.3 Generalized Confidence Interval Method (GCI)

Weerahandi [12] provided a general method for obtaining a confidence interval based on a generalized pivotal quantity. The generalized pivotal quantity is defined as a special pivotal quantity [23], essentially having two properties. First, the probability distribution of the pivotal quantity is free of any unknown parameters, and second, the observed pivot is also independent of unknown parameters. The generalized pivot for ρ is given by [18],

(11) $$g(t)=\frac{max\left[0,\frac{(n-1)MSS}{k\chi_{n-1}^{2}}-\frac{(n-1)(k-1)MSE}{k\chi_{\left(n-1)(k-1\right)}^{2}}\right]}{\frac{(k-1)MSR}{n\chi_{k-1}^{2}}+\frac{(n-1)MSS}{k\chi_{n-1}^{2}}+\frac{(nk-n-k+1)(nk-n-k)MSE}{nk\chi_{\left(n-1)(k-1\right)}^{2}}},$$

where t denotes a vector of mean squares. The (1−α)×100% confidence interval for ρ is given by the quantile function Ĝ as [11, 16, 18]

$$\begin{array}{l} L,U=Ĝ(\frac{\alpha }{2}),Ĝ(1-\frac{\alpha }{2}) \end{array}$$

We denote this method henceforth as GCI. Unlike the other confidence interval methods, the generalized confidence interval method relies on empirical quantiles of the pivotal quantity that are obtained through sampling methods. Specifically, this is achieved by sampling from the $\chi^{2}$‐distributions in Equation (11) and plugging in the values obtained [11, 16]. This is then repeated a large number of times, implying that the accuracy of this method relies on the number of simulations. Dobbin and Ionan [18] state that if the confidence interval is obtained properly (i.e., using the recommended 100 000 simulations or more [24]), then the difference between Ĝ and G is negligible. Note that the third term of the denominator of g(t) is incorrect in Ionan et al. (see Appendix C for the correction of the formula in the paper of Ionan et al. [11]).

### 3.4 Variance Partitioning Method ($VP_{F}$, $VP_{B}$)

Demetrashvilli et al. [13] proposed two generic approaches to construct a confidence interval for an ICC coefficient for a general ANOVA model with any number of variance components. This can be applied to the ICC for agreement for the two‐way ANOVA model. These approaches rely on the partitioning of the relevant variance components into two parts. Consider that there are a total of P variance components ($\sigma_{1}^{2},\sigma_{2}^{2},\ldots ,\sigma_{Q}^{2},\sigma_{Q+1}^{2},\ldots ,\sigma_{P}^{2}$) which can be divided into two partitions such that,

1. the first partition q=1,…,Q of the variance components $\sigma_{1}^{2},\ldots ,\sigma_{Q}^{2}$ is unrelated to the rater process, the sum of which is denoted as $\sigma_{G}^{2}$, and
2. the second partition p=Q+1,…,P of the variance components $\sigma_{Q+1}^{2},\ldots ,\sigma_{P}^{2}$ represents part of the rater variability, the sum of which is denoted as $\sigma_{E}^{2}$.

Then ρ Equation (2) can be defined in a general form, $\rho_{G}=\frac{\sigma_{G}^{2}}{\sigma_{G}^{2}+\sigma_{E}^{2}}$, where $\sigma_{G}^{2}=\sum_{q=1}^{Q}\sigma_{q}^{2}$ and $\sigma_{E}^{2}=\sum_{p=Q+1}^{P}\sigma_{p}^{2}$. For the two‐way ANOVA Equation (1), P=3, Q=1, $\sigma_{G}^{2}=\sigma_{s}^{2}$ and $\sigma_{E}^{2}=\sigma_{e}^{2}+\sigma_{r}^{2}$. Denoting the total variance $\sigma_{T}^{2}=\sigma_{s}^{2}+\sigma_{E}^{2}$, the estimated variance of the estimator of ρ is [13]

(12) $$\begin{array}{l} \hat{V}(\hat{\rho })\approx \frac{{\hat{\sigma }}_{E}^{4}}{{\left({\hat{\sigma }}_{T}^{2}\right)}^{4}}\hat{V}\left({\hat{\sigma }}_{s}^{2}\right)+\frac{{\hat{\sigma }}_{s}^{4}}{{\left({\hat{\sigma }}_{T}^{2}\right)}^{4}}\hat{V}\left({\hat{\sigma }}_{E}^{2}\right)-\frac{2{\hat{\sigma }}_{s}^{2}{\hat{\sigma }}_{E}^{2}}{{\left({\hat{\sigma }}_{T}^{2}\right)}^{4}}\hat{Cov}\left({\hat{\sigma }}_{s}^{2},{\hat{\sigma }}_{E}^{2}\right), \end{array}$$

where $\hat{V}({\hat{\sigma }}_{E}^{2})=\hat{V}({\hat{\sigma }}_{r}^{2})+\hat{V}({\hat{\sigma }}_{e}^{2})+2\hat{Cov}({\hat{\sigma }}_{r}^{2},{\hat{\sigma }}_{e}^{2})$ and $\hat{Cov}({\hat{\sigma }}_{i}^{2},{\hat{\sigma }}_{j}^{2})$ is the covariance of the estimators ${\hat{\sigma }}_{i}^{2}$ and ${\hat{\sigma }}_{j}^{2}$. Unlike the other confidence interval methods, the covariance between the variance estimators is required to obtain $\hat{V}(\hat{\rho })$. The variance‐covariance estimates of the variance components are then obtained from the inverse of the Fisher information matrix, as provided in Searle et al. [25] (Chapter 4, page 155). One can use Equation (12) to construct a confidence interval for ρ using the Wald confidence interval relying on a normal approximation. However, the distribution of $\hat{\rho }$ is generally skewed. Therefore, instead of a normal distribution, Demetrashvilli et al. [13] proposed using an F‐ or beta‐distribution to approximate the distribution of $\hat{\rho }$, which we denote by the F‐ and Beta‐methods, respectively.

#### 3.4.1 F‐Method ($VP_{F}$)

The F‐method is based on Satterthwaite's approximation of the variance components $\sigma_{s}^{2}$ and $\sigma_{E}^{2}$, where the estimate of the two components follow independent $\chi^{2}$‐distributions with the degrees of freedom $df_{1}$ and $df_{2}$ estimated as ${\hat{df}}_{1}=\frac{2{\hat{\sigma }}_{s}^{4}}{\hat{V}({\hat{\sigma }}_{s}^{2})}$ and ${\hat{df}}_{2}=\frac{2{\hat{\sigma }}_{E}^{4}}{\hat{V}({\hat{\sigma }}_{E}^{2})}$, respectively.

In the study of van den Heuvel [26], it was found that the F‐method works better when the variance estimates do not include covariance terms. Then $\hat{\rho }$ is distributed according to,

$$\begin{array}{l} \frac{{\hat{\sigma }}_{s}^{2}f_{{\hat{df}}_{1}{\hat{df}}_{2}}}{{\hat{\sigma }}_{s}^{2}f_{{\hat{df}}_{1}{\hat{df}}_{2}}+{\hat{\sigma }}_{E}^{2}}, \end{array}$$

where $f_{\nu_{1},\nu_{2}}$ denotes the density of an F‐distribution with $\nu_{1}$ and $\nu_{2}$ degrees of freedom in the numerator and denominator, respectively. The (1−α)×100% confidence interval for ρ is given by,

 L=σ^s2Fdf^1,df^2(α2)σ^s2Fdf^1,df^2(α2)+σ^E2and U=σ^s2Fdf^1,df^2(1−α2)σ^s2Fdf^1,df^2(1−α2)+σ^E2.

Note that, Demetrashvilli et al. [13] suggests to use ${\hat{df}}_{1}=max(1,\frac{2{\hat{\sigma }}_{s}^{4}}{\hat{V}({\hat{\sigma }}_{s}^{2})})$ to avoid small values of ${df}_{1}$ which might be computationally complex.

#### 3.4.2 Beta‐Method ($VP_{B}$)

The Beta‐method [13] aims to approximate the distribution of $\hat{\rho }$ with a beta‐distribution with parameters a>0 and b>0. The parameters of the beta‐distribution are obtained by matching the theoretical mean and variance of the beta‐distribution to the mean and variance of $\hat{\rho }$ to obtain,

(13)  a^=ρ^[ρ^(1−ρ^)−V^(ρ^)]V^(ρ^) and b^=(1−ρ^)[ρ^(1−ρ^)−V^(ρ^)]V^(ρ^)

Note that the estimation of these parameters a and b may lead to several complications, and Demetrashvilli et al. [13] suggest the following two corrections to avoid these problems.

1. A beta‐distribution is U‐shaped when both a and b are less than 1 (a,b<1). This shape is not appropriate to approximate the distribution of $\hat{\rho }$. Therefore, to avoid parameter estimates $â,\hat{b}<1$, two fixes are depending on whether $\hat{\rho }$ is greater than or less than 0.5.
  - When $0<â,\hat{b}<1$ and $\hat{\rho }=\frac{â}{â+\hat{b}}<0.5$, the U‐shaped beta‐distribution is converted to a J‐shaped beta‐distribution, by increasing the mass closer to 0. This is done by fixing $\hat{b}=1$ and leaving the other parameter â<1 (estimated by Equation 13).
  - When $0<â,\hat{b}<1$ and $\hat{\rho }=\frac{â}{â+\hat{b}}>0.5$, the U‐shaped beta‐distribution is converted to a J‐shaped beta‐distribution by increasing the mass closer to 1. This is done by fixing â=1 and leaving the other parameter $\hat{b}<1$ (estimated by Equation 13).
2. The estimates for a and b from Equation (13) may be negative when the variance of $\hat{\rho }$ Equation (12) is larger than $\hat{\rho }(1-\hat{\rho })$. Since a beta‐distribution is defined for positive parameters, two fixes are proposed in this case, depending on whether $\hat{\rho }$ is greater or lesser than 0.5, for the same reasons mentioned above.
  - When $\hat{\rho }\leq 0.5$, $\hat{b}$ is fixed at 1 ($\hat{b}=1$) and â is obtained as $â=\hat{\rho }/(1-\hat{\rho })$.
  - When $\hat{\rho }>0.5$, â is fixed at 1 (â=1) and $\hat{b}$ is obtained as $\hat{b}=\hat{\rho }/(1-\hat{\rho })$.

Based on this approximate distribution, the (1−α)×100% confidence interval for ρ is given by,

$$\begin{array}{l} L=B_{â,\hat{b}}\left(\frac{\alpha }{2}\right)\;,\;\text{and}\;U=B_{â,\hat{b}}(1-\frac{\alpha }{2})\;, \end{array}$$

where $B_{â,\hat{b}}(\frac{\alpha }{2})$ and $B_{â,\hat{b}}(1-\frac{\alpha }{2})$ are the $(\frac{\alpha }{2})\times 100$ and $(1-\frac{\alpha }{2})\times 100$ percentiles of a beta‐distribution with parameters â and $\hat{b}$.

## 4 Comparison of Confidence Interval Methods

Several comparative studies [5, 8, 11, 14, 15, 16] investigated the statistical properties of the confidence interval methods reviewed in this paper. These studies are simulation based and compare the coverage probability and the average width of the different confidence interval methods for ρ. Coverage probability is defined as the proportion of confidence intervals from the simulations that contain the true value of ρ. The average width of the confidence interval is the difference between the upper and lower limits of the confidence interval, averaged over the simulations. A confidence interval method with coverage probability close to the nominal level and having the smallest average width is considered to be the best.

The choice of the confidence interval methods for our comparison is motivated by the results from the previous studies. Specifically, Almehrizi and Emam [8] demonstrated that their matrix formulation method outperformed the two‐moment approximation method. Zou and McDermott [5] compared all the moment approximation methods, but could not recommend one method over the others. Rousson et al. [14] and Cappelleri and Ting [15] compared some of the better moment approximation methods with the modified large sample methods, leading to the conclusion that the latter methods are better. Ionan et al. [11] and Tian and Cappelleri [16] compared the modified large sample method proposed by Gui et al. [10] with the generalized confidence interval method, but suggested that neither of these methods is better than the others in all scenarios (i.e, combinations of ρ, R, k, and n). Xiao and Liu [6] compared their modified profile likelihood method with the generalized confidence interval method and showed that their method produces over‐coverage in most cases. These comparisons are limited as, first, they do not encompass all the available confidence interval methods, and second, the scenarios in which these comparisons are made vastly differ across papers. For example, the matrix formulation was only compared to the two‐moment approximation method, whereas the moment approximation methods have been shown to be inferior to several other confidence interval methods, like the modified large sample methods and the generalized confidence interval method. Therefore, we have included these methods (matrix formulation of $\hat{\rho }$, modified large sample methods, generalized confidence interval method) in our comparison. Further, the variance partitioning method, not included in previous comparisons, is added to our comparative study. The modified profile likelihood method is excluded from our study mainly for two reasons. First, the modified profile likelihood relies on a specification of a plausible range for ρ and R, which is generally unavailable in a reliability study. Second, this method uses two consecutive grid searches combined with Monte‐Carlo simulations, implying a high computational cost of the method.

In summary, we compare the coverage probability and the average width of the delta method applied to the log‐transformation and the matrix formulation of $\hat{\rho }$ ($W_{log}$ and $W_{mat}$, respectively), the modified large sample methods by Arteaga et al. [9] and Gui et al. [10] ($MLS_{A}$ and $MLS_{G}$, respectively), the generalized confidence interval method (GCI) and the variance partitioning methods using the F‐ and Beta‐methods ($VP_{F}$ and $VP_{B}$, respectively).

### 4.1 Monte Carlo Simulation

We set up a Monte Carlo simulation study to evaluate the statistical properties of the seven confidence interval methods. Following the ANOVA model defined in Equation (1), for each replication, N=n×k errors are drawn from a normal distribution centered at zero with variance $\sigma_{e}^{2}=1$. Then, for a given value of R, k rater effects ($r_{j}$) are drawn for n participants following a normal distribution centered in zero with variance $\sigma_{r}^{2}=R\times \sigma_{e}^{2}=R$ (since $\sigma_{e}^{2}=1$). Next, for a given value of ρ, n participant effects ($s_{i}$) are drawn for k raters from a normal distribution centered in zero with variance $\sigma_{s}^{2}=\frac{\rho (R+1)}{1-\rho }$. This process is replicated $10^{5}$ times. For each replication, the confidence interval using the above‐mentioned seven confidence interval methods is obtained. We study the properties of these confidence interval methods for various scenarios. Specifically, we choose n∈{20,40,60,100}, which covers a wide range of the number of participants; ρ∈{0.7,0.8,0.9}, since a high ρ is generally of interest in a reliability study; and, R∈{0.1,1,2} based on empirical evidence from several reliability studies [18, 27, 28, 29, 30, 31, 32, 33, 34, 35] which indicate that in most cases R≤1. As for the number of raters, we choose k∈{2,3,5,8}. Two‐sided confidence intervals are commonly used for sample size determination procedures (see Section 5). Therefore, we compare the obtained two‐sided confidence intervals based on the coverage probability as well as the average width of the confidence interval. Confidence interval methods providing a coverage probability closer to the nominal coverage are more accurate, thus, we examine how far the coverage probability is from the nominal coverage. For a 95% confidence interval, a distance smaller than 0.01 (i.e., the coverage probability is between 0.94 and 0.96) is considered to be the best, whereas a distance larger than 0.05 (i.e., the coverage probability is below 0.90) is considered to be the worst. As for the width of the confidence interval, the average difference between the limits of the confidence interval is obtained. Since a shorter width of the confidence interval is desirable, among methods that have a coverage probability close to the nominal coverage probability (e.g., a distance less than 0.01 from 0.95 for a 95% confidence interval), a method with a smaller average width is preferred.

### 4.2 Results of the Coverage Probability

**FIGURE 1:**
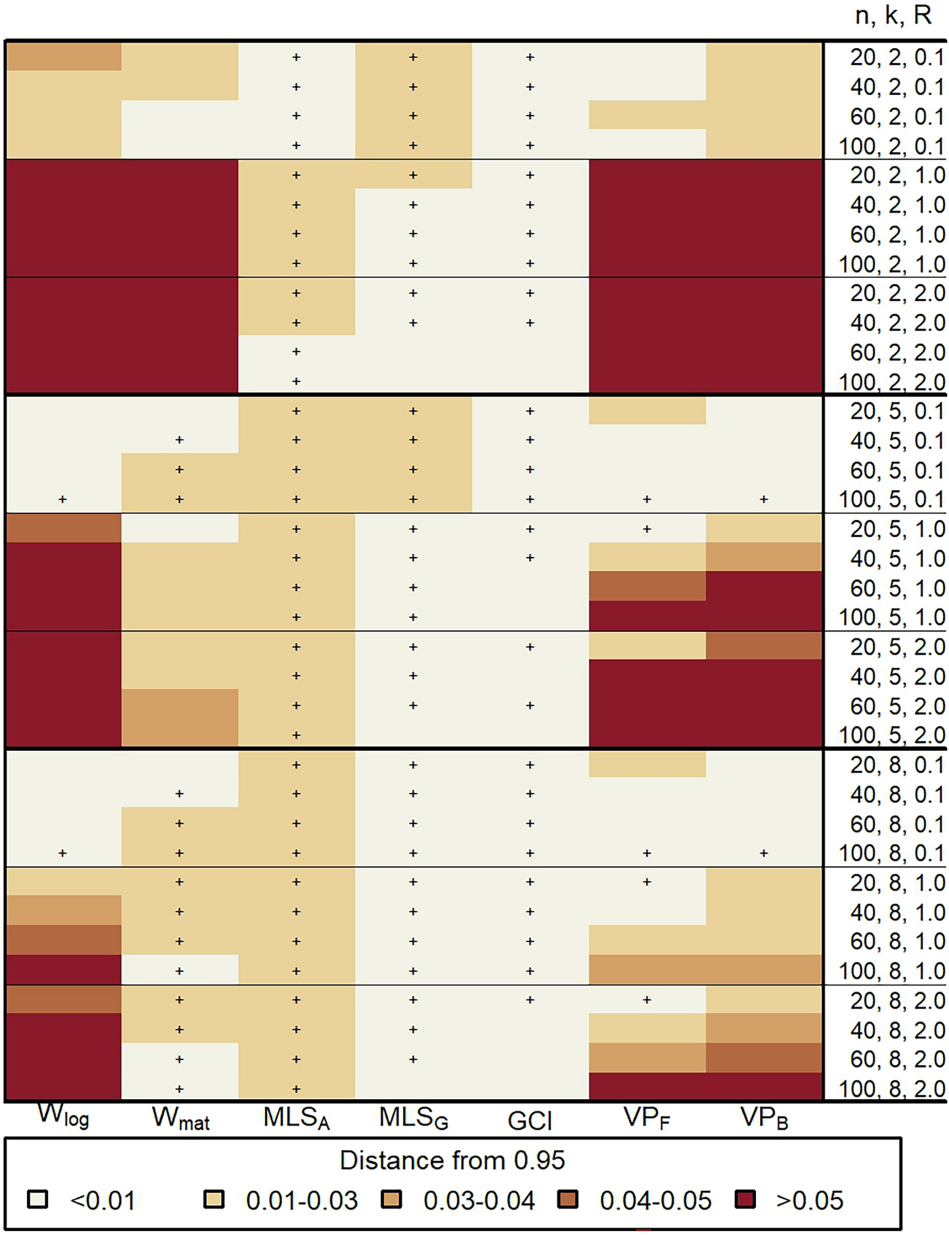
Heatmap depicting the empirical coverage probability from the Monte Carlo simulation study for the 95% two‐sided confidence interval for ρ=0.7. The distances from 0.95 are color‐graded with a $“{+}^{\prime \prime }$ sign denoting over‐coverage. A distance ≤0.01 indicates that the coverage probability is within the interval [0.94,0.96] with a $“{+}^{\prime \prime }$ sign denoting coverage probability within [0.95,0.96]; a distance (0.01,0.03] indicates that the coverage probability is within [0.92,0.94) or (0.96,0.98] ($“{+}^{\prime \prime }$ sign); a distance (0.03,0.04] indicates that the coverage probability is within [0.91,0.92) or (0.98,0.99] ($“{+}^{\prime \prime }$ sign); a distance (0.04,0.05] indicates that the coverage probability is within [0.90,0.91) or (0.99,1.00] ($“{+}^{\prime \prime }$ sign) and a distance >0.05 indicates that the coverage probability is <0.90.

We start by presenting the coverage probability results of the Monte Carlo simulation study. Since the coverage probability generally improves as ρ increases, Figure 1 summarizes the results for a two‐sided 95% confidence interval with ρ=0.7, which is the lowest value of ρ in our study. Complete results can be found in Supporting Information Table S1. The coverage probability of the confidence intervals is color‐graded based on how far it is from 0.95. To indicate when the coverage probability of the confidence interval is greater than 0.95, we have marked it with a plus sign. The figure shows that the confidence interval method GCI provides a coverage probability between 0.94 and 0.96 in all scenarios. This is closely followed by $MLS_{G}$, which provides coverage probability within the interval [0.94,0.96] in many cases. In other cases, for example, when R=0.1 and k≤5, $MLS_{G}$ provides over‐coverage with a coverage probability within the interval (0.96,0.98]. Conclusions on under‐ or over‐coverage can be drawn from the complete results in Supporting Information Table S1. The coverage probability of the other methods varies depending on the scenario. The general trends are discussed below on a case‐by‐case basis.

When k=2 and R=0.1, the confidence interval method $MLS_{A}$ and $VP_{F}$ provide coverage within the interval [0.94,0.96] for n≥20, while $VP_{B}$ shows under‐coverage [0.92,0.94) for n≥20. $W_{mat}$ performs badly for small values of n and provides a coverage within the interval [0.94,0.96] when n≥60. $MLS_{A}$, $VP_{F}$, $VP_{B}$ and $W_{mat}$ also provide similar coverage for 0<R<0.1 (results not shown). As R increases, the performance of the methods generally worsens. Specifically, $W_{log}$, $W_{mat}$, $VP_{F}$, and $VP_{B}$ show significant under‐coverage with a coverage below 0.70 (see Supporting Information Table S1). When R=1, $MLS_{A}$, however, shows over‐coverage with a coverage within (0.96,0.98], and its performance improves as R increases (for details, see Supporting Information Table S1).

Summarizing the results for k>2, $MLS_{A}$ provides a coverage within the interval (0.96,0.98] for R≤2 and n≥20, and improves to a coverage within [0.94,0.96] as R increases. As for the remaining methods, when R=0.1, $W_{log}$ and $VP_{B}$ provide a coverage within [0.94,0.96] for n≥20. For the same R, $VP_{F}$ exhibits a coverage within [0.94,0.96] when n≥40. $W_{mat}$ provides a coverage within [0.94,0.96] when n≤40 and over‐coverage with a coverage within (0.96,0.98] when n≥60. Increasing R, however, has a detrimental effect on the coverage probabilities of $W_{log}$, $W_{mat}$, $VP_{F}$, and $VP_{B}$. For example, when k=8 and n=100, the coverage probability of $W_{log}$ decreases from within [0.94,0.96] for R=0.1 to less than 0.88 for R=2 (see Supporting Information Table S1).

### 4.3 Results of the Average Width

**FIGURE 2:**
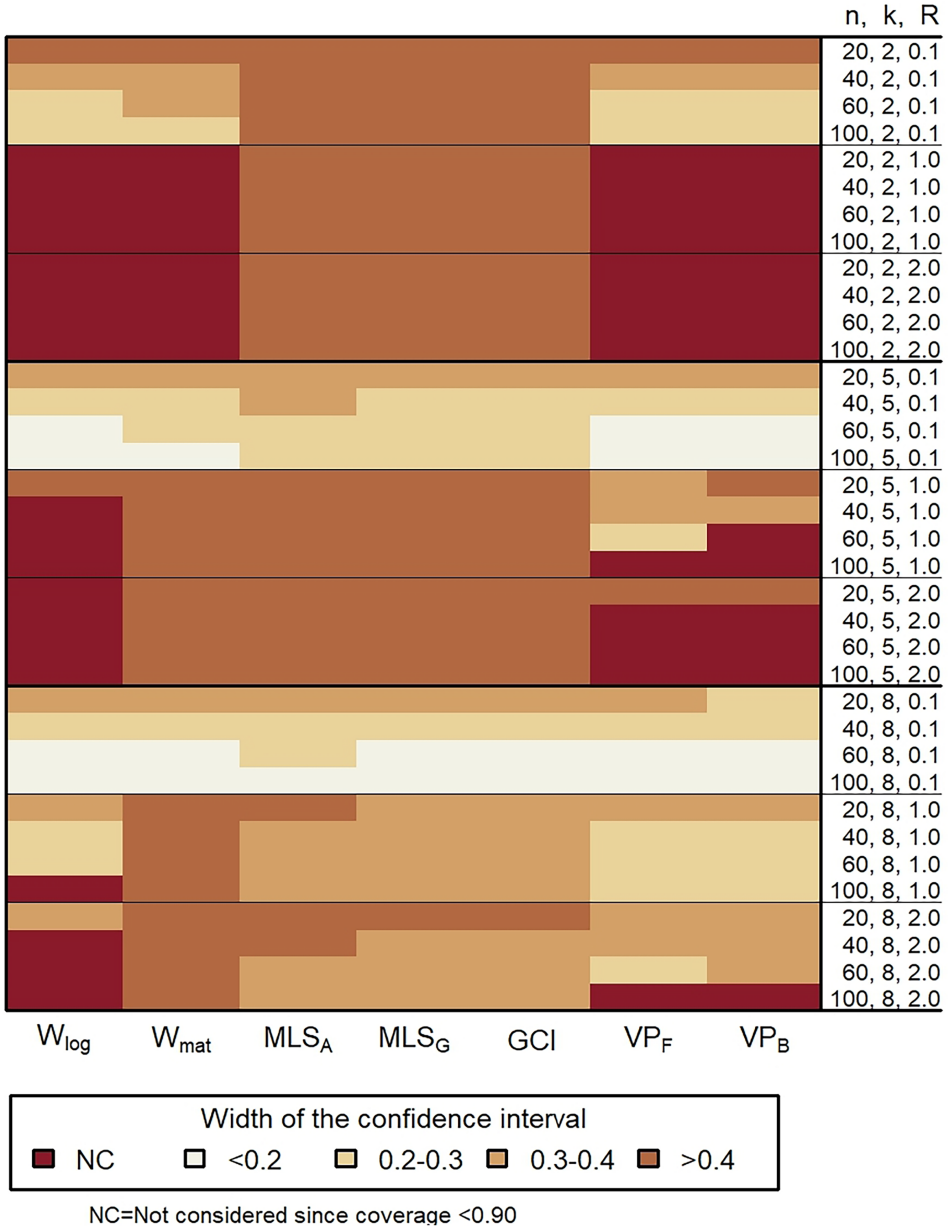
Heatmap depicting the results of the average width of the confidence interval in the Monte Carlo simulation study for the 95% two‐sided confidence interval for ρ=0.7. The average width of the confidence interval is not considered (NC) when the coverage probability was <0.90 (see Figure 1).

As for the width of the confidence interval, similar to the coverage probability, we chose to present the results for ρ=0.7, as it represents the worst case scenario. The width of the confidence intervals becomes smaller as ρ increases (see Supporting Information Table S1). Figure 2 summarizes the results of the average width from the Monte Carlo simulation study for the 95% two‐sided confidence interval for ρ=0.7. Generally, keeping n, k, and ρ constant, as R increases, the average width of the confidence interval increases, which was also observed in previous studies [11, 15]. Furthermore, as expected, increasing k while keeping n, R, and ρ constant, the average width of the confidence interval decreases. From Figure 2 it can be seen that $VP_{F}$ provides a shorter average width of the confidence interval in most cases. It is important to note that although GCI provides a coverage within [0.94,0.96] in all scenarios studied (see Figure 1), the width of the confidence interval obtained using this method is quite large (see Figure 2). For example, when R=0.1, k=2 and n=40, the width of the confidence interval for GCI is about twice as large as that of $VP_{F}$ (see Supporting Information Table S1). However, $VP_{F}$ provides a coverage within [0.94,0.96] almost only for R≤0.1. Nonetheless, as k increases, for example, when k=8, the average width of the confidence interval for GCI and $VP_{F}$ is comparable, especially for n≥40. Note that the average width of $MLS_{G}$ and GCI were shown to be asymptotically equivalent [18], and this is also observed in Figure 2.

### 4.4 Remarks and Guide to Choose a Confidence Interval Method

When selecting a method for constructing a confidence interval for ρ, it is essential to consider several factors. Figures 1 and 2 provide insights into the performance of the different methods in terms of coverage probability and confidence interval width under various scenarios. The figures show that the number of raters, k, and the rater‐to‐error variance ratio, R, play a major role in the performance of the methods.

From Figures 1 and 2, it is evident that no method produces a confidence interval width smaller than 0.2 when R≥1. However, two critical points should be considered. First, we expect the values of R to be smaller than 1 in many applications [18, 27, 28, 29, 30, 31, 32, 33, 34, 35], which results in acceptable widths of the confidence intervals and acceptable coverage probabilities. Second, the results presented in this section are for ρ=0.7, which corresponds to the worst‐case scenario in our study. Reliability studies typically target a larger value of ρ (ρ≥0.7). As ρ increases, the width of the confidence interval decreases for all methods, and the coverage probability approaches the nominal coverage for most confidence interval methods, leading to acceptable performance (for details see Supporting Information Table S1).

Table 2 summarizes the results for ρ≥0.7. When the number of raters is large (e.g., k≥8), then we recommend the use of GCI or $MLS_{G}$. When the number of raters is small (e.g., k<8), we recommend the use of $VP_{F}$ when R≤0.1, and again of GCI or $MLS_{G}$ when R>0.1. Since during the planning stage of a reliability study R is commonly unknown, when one is uncertain about the value of R or when R is known and R>0.1, $MLS_{G}$ and GCI are the only reasonable options. Notably, no method appears to yield a width of the confidence interval less than 0.2 when R≥1.

**TABLE 2:** Recommendations for confidence interval method combining the results of the coverage probability and average width of the different confidence interval methods.

	R≤0.1	R>0.1
k<8	$VP_{F}$	$MLS_{G}$, GCI, ($MLS_{A}$ when R≥2)
k≥8	$MLS_{G}$, GCI	$MLS_{G}$, GCI

## 5 Sample Size Determination for an Expected Width of the Confidence Interval

The number of raters/participants can be determined based on the expected width of the confidence interval approach [36], which utilizes the different confidence interval construction methods discussed in Section 3. The approach searches for the minimum number of participants, n and/or raters, k, such that the expected width of a confidence interval (W) for a planned ICC value, ρ, is approximately equal to or less than a constant ω. We regard the resulting n and k as the optimal combination of n and k. Note that this approach depends on a planned ICC value, ρ, and the rater‐to‐error variance ratio R ($=\sigma_{r}^{2}/\sigma_{e}^{2}$) and thereby yields valid results when the initial guess for ρ and R is accurate.

**FIGURE 3:**
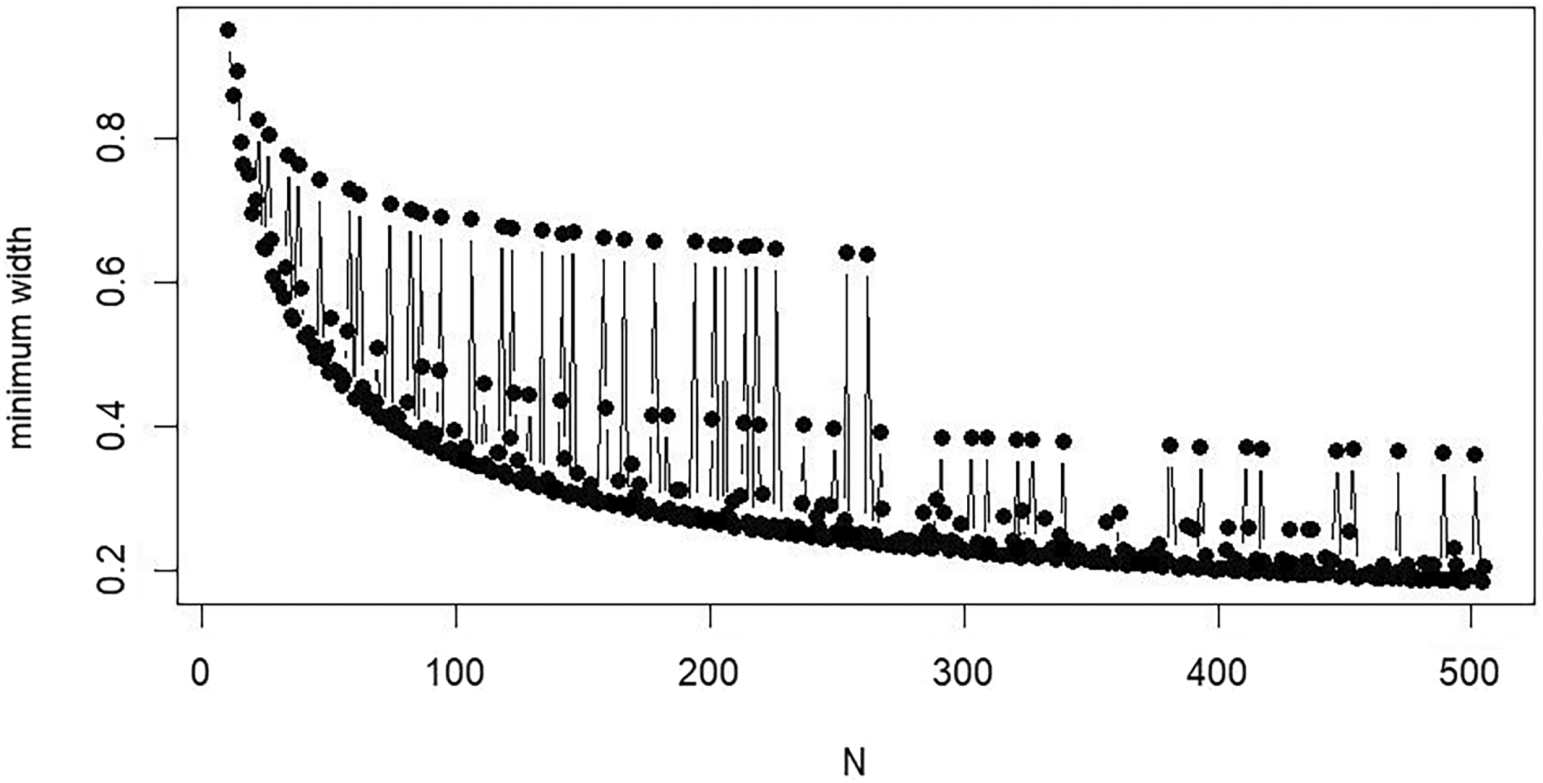
Plot of the minimum width of the confidence interval of $MLS_{G}$ obtained using the combinations of n and k according to the procedure of Saito et al. [7] for ρ=0.7, R=0.1, and N∈{10,505}.

In the literature, three procedures to determine sample sizes based on the expected width of the confidence interval can be identified. These procedures determine the optimal combination of n and k by searching through possible nodes of the grid for n and k. Saito et al. [7] proposed a procedure that utilizes an analytical formula to search through a small number of nodes in the grid to identify the optimal combination. While using an analytical formula avoids searching through all possible nodes of the grid for n and k, the procedure has two significant drawbacks that limit its practical use. The procedure starts with an arbitrary value of N=n×k where the combination of n and k, for a given N, is determined using an analytical formula (see Equation 5 in Supporting Information Data S1), which may produce non‐integer values. Since integer values for n and k are required, combinations of n and k near the calculated values are obtained. The width of the confidence interval for these combinations is then determined via simulations. If the confidence interval width is not sufficiently small or is much smaller than ω, the previous steps are repeated by increasing or decreasing N, respectively. However, when increasing N, the minimum width of the confidence interval for an integer combination of n and k may not necessarily decrease, as exemplified in Figure 3, where the width is determined using the $MLS_{G}$ method. A plausible solution is to iteratively determine the confidence interval width by increasing N from the minimum feasible value and checking for each N whether the width is less than ω. However, this significantly increases the computation time required for the procedure and is infeasible if the range of N is not small enough (e.g., if it exceeds three or four values). Another drawback is that, since the procedure relies on specifying a range for the total number of observations N, it may result in a combination of n and k that is not feasible in practice. The details of the procedure are given in Supporting Information Data S1 (Section B), but this procedure is not considered further in our evaluation.

Doros and Lew [17] proposed a general procedure that is purely simulation‐based and searches through all possible nodes of n and k to identify the optimal combination. This procedure also requires extensive simulations to evaluate the width of the confidence interval for each combination of n and k. Consequently, it can be rather time‐consuming and inefficient. However, this procedure can be adapted by fixing k to a limited set of feasible values, thus reducing the computational burden. Finally, Dobbin and Ionan [18] developed a procedure that allows either n or k to be fixed. By using analytical formulas for the expected width of the confidence intervals for GCI and also $MLS_{A}$, this procedure has the advantage of having the shortest computation time. However, since the analytical results cannot be easily extended to other confidence interval methods, this procedure is also the least general. These two procedures are described in detail in the following subsections.

### 5.1 Purely Simulation‐Based Procedure

Doros and Lew [17] proposed a purely simulation‐based procedure for finding the optimal combination of n and k based on the expected width of the confidence interval. The procedure entails:

1. Simulate $y_{ij}$ according to Equation (1), and calculate the mean squares as defined in Table 1;
2. Estimate the expected width of the confidence interval by averaging over a large number of simulations for a variety of n and k values;
3. Return the combination of n and k which results in an expected width of the confidence interval smaller than or approximately equal to the predefined value (ω).

**FIGURE A1:**
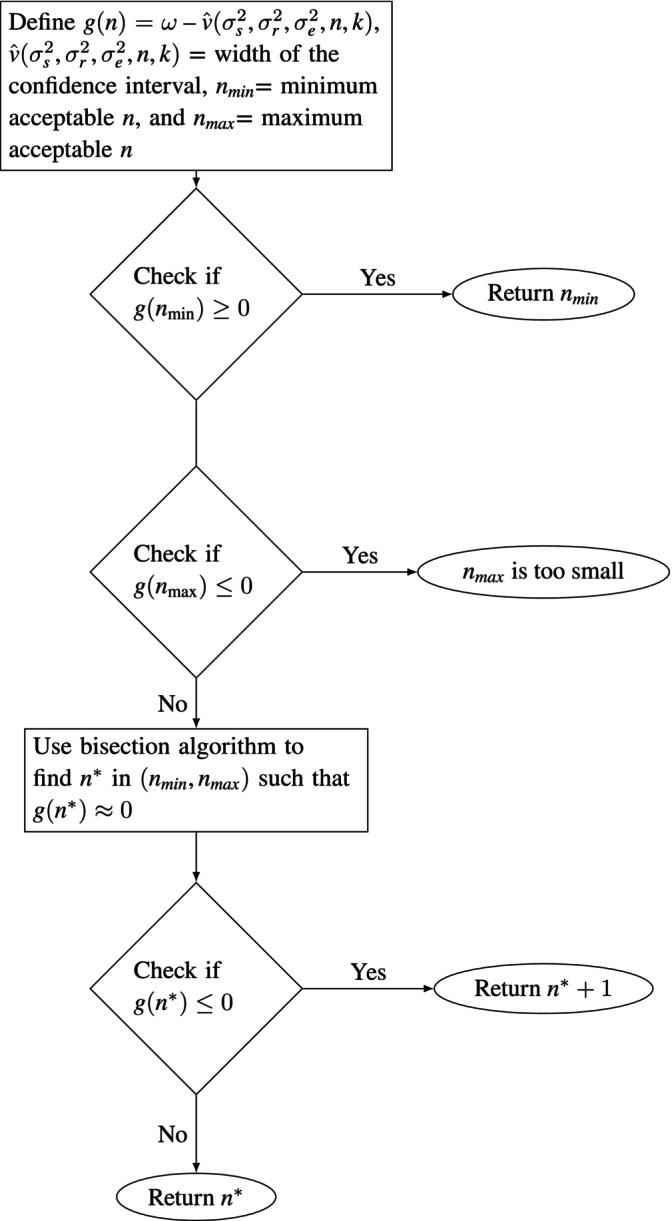
The steps of the algorithm to obtain a sample size of participants, n for a pre‐specified expected width of the confidence interval ω, a number of raters, k, a planned value for the ICC for agreement, ρ and the rater‐to‐error variance ratio, R.

Doros and Lew [17] proposed this sample size determination procedure for the two‐moment approximation method, but it may be combined with the other confidence interval methods. To reduce the computational cost of this procedure, and because the search can result in a combination of n and k not feasible in practice, the grid search can be restricted by fixing either n or k. Considering a limited range of values for k, and utilizing the confidence interval methods $VP_{F}$ and $MLS_{G}$, we implemented an algorithm to obtain the minimum number of participants, $n^{*}$ ensuring that the expected width of the confidence interval remains below a pre‐specified value. For each k, the corresponding number of participants $n^{*}$ is determined using the bisection algorithm, and the steps of the algorithm are provided in Figure A1 in Appendix A. Note that this procedure is very time‐consuming when combined with the GCI method. When the confidence interval width is obtained according to GCI, the procedure proposed by Dobbin and Ionan [18] is preferable (see Section 5.2).

Table 3 shows sample sizes obtained for the confidence interval method $VP_{F}$ and $MLS_{G}$ using the procedure proposed by Doros and Lew [17] by fixing k∈{5,10,15}, ρ∈{0.7,0.8,0.9}, and R∈{0.1,2} such that the expected width of the confidence interval is less than ω, with ω∈{0.2,0.3}. The width of the confidence interval was obtained from $10^{4}$ Monte Carlo simulations using a two‐sided 95% confidence interval. Sample sizes for $VP_{F}$ were not calculated for R=2 since when R=2, we found the coverage probability for $VP_{F}$ to be unacceptable (see Section 4.2). As for $MLS_{G}$, it can be further observed from Table 3 that for some scenarios the sample size determination procedure did not return a finite value (n≤500), denoted by the blanks in the table. This probably occurs since the asymptotic width of $MLS_{G}$ for the given R, k, and ρ is greater than ω. Comparing the sample sizes for $VP_{F}$ and $MLS_{G}$, it can be observed that sample sizes for $MLS_{G}$ are rather close to those of $VP_{F}$ when R=0.1 and k≥10. It can be further seen from Table 3 that as k or ρ increase, a smaller n is required to achieve the same ω. However, the decrease in n with an increase in k is not as large when k increases from 10 to 15, compared to when k increases from 5 to 10.

**TABLE 3:** Sample sizes obtained with the procedure of Doros and Lew [17] using the expected width of the two‐sided 95% confidence interval for the method $VP_{F}$ and $MLS_{G}$, for ρ∈{0.7,0.8,0.9}, R∈{0.1,2}, k∈{5,10,15}, and ω∈{0.2,0.3}. The width of the confidence interval is based on $10^{4}$ Monte Carlo simulations. No sample size was obtained for $VP_{F}$ for R=2, since when R=2, we found the coverage probability for $VP_{F}$ to be unacceptable (see Section 4.2). The blanks for the sample size for $MLS_{G}$ indicate that a width of the confidence interval less than ω could not be obtained with n≤500.

			ω=0.2		ω=0.3	
R	k	ρ	$VP_{F}$	$MLS_{G}$	$VP_{F}$	$MLS_{G}$
0.1	5	0.7	53	85	28	33
		0.8	34	42	16	20
		0.9	15	15	8	7
	10	0.7	45	45	20	18
		0.8	27	27	14	14
		0.9	14	11	8	7
	15	0.7	40	39	19	18
		0.8	25	22	13	11
		0.9	12	11	8	5
2	5	0.7				
		0.8				
		0.9				
	10	0.7				114
		0.8				19
		0.9		19		7
	15	0.7				32
		0.8		89		17
		0.9		17		5

### 5.2 Sample Size Determination Procedure According to Dobbin and Ionan [18]

The sample size calculation proposed by Dobbin and Ionan [18] is based on the average width of the confidence interval according to $MLS_{A}$ and GCI. Note that Dobbin and Ionan [18] refer through their paper to the paper of Cappelleri and Ting [15], who only discussed $MLS_{G}$, which could be misleading. Denoting the width of the confidence intervals obtained with the $MLS_{A}$ and GCI methods as ${\hat{v}}_{MLS_{A}}(t)$ and ${\hat{v}}_{GCI}(t)$, the average widths are,

 μMLSA(q)=E[v^MLSA(t)|q] and μGCI(q)=E[v^GCI(t)|q]

where $q=(\sigma_{s}^{2},\sigma_{r}^{2},\sigma_{e}^{2},n,k)=(\rho ,R,n,k)$, that is, the unknown quantities. The sample size determination procedure using the generalized confidence interval method (Section 3.3) aims to find n when k is fixed, by finding the minimum value of n with an expected width smaller than ω, or, similarly, the minimum value of k, when n is fixed. When the modified large sample confidence interval is used, $\mu_{GCI}$ is replaced by $\mu_{MLS_{A}}$ in the above criterion. Dobbin and Ionan [18] further derived the asymptotics (see Appendix B), which enables one to check whether a width of the confidence interval is achievable under the parameters specified. When the width is achievable, Dobbin and Ionan [18] showed that the functions $\mu_{MLS_{A}}$, and $\mu_{GCI}$ can be obtained numerically via Monte Carlo simulations.

#### 5.2.1 Estimating $\mu_{MLS_{A}}(q)$

A novel procedure referred to as “dependent conditioning” was developed by Dobbin and Ionan [18] to obtain $\mu_{MLS_{A}}$ which can be summarized in the following four steps.

1. The limits of the confidence interval U, L and their difference U−L are written in terms of two F‐distributed random variables, $X_{SE}(=\frac{MSS/(k\sigma_{s}^{2}+\sigma_{e}^{2})}{MSE/\sigma_{e}^{2}})$ and $X_{RE}(=\frac{MSR/(n\sigma_{r}^{2}+\sigma_{e}^{2})}{MSE/\sigma_{e}^{2}})$.
2. The bivariate joint density of $X_{SE}$ and $X_{RE}$ is obtained, after which the conditional density, $f(x_{RE}|x_{SE})$ is calculated.
3. The variable $X_{RE}$ is integrated out of the conditional density by expressing analytically the integral using a hypergeometric function [18].
4. The final width estimator is obtained using Monte Carlo simulations of a single variable following $F_{n-1,(n-1)(k-1)}$. The width estimator is given as
$$\begin{array}{l} {\hat{v}}_{MLS_{A}}=\frac{1}{M}\sum_{m=1}^{M}\left(E[U|{\left(x_{SE}\right)}_{m}]-E[L|{\left(x_{SE}\right)}_{m}\right), \end{array}$$
  where M is the total number of Monte Carlo simulations and ${\left(x_{SE}\right)}_{m}$ are independent and identically distributed draws from $F_{n-1,(n-1)(k-1)}$. The analytic expressions for $E[U|{\left(x_{SE}\right)}_{m}]$ and $E[L|{\left(x_{SE}\right)}_{m}]$ are obtained using hypergeometric functions, or can be obtained numerically using Monte Carlo simulations [18].

#### 5.2.2 Estimating $\mu_{GCI}(q)$

The process of obtaining $\mu_{GCI}$ requires obtaining ${\hat{v}}_{GCI}(t)$. Unlike ${\hat{v}}_{MLS_{A}}(t)$, obtaining ${\hat{v}}_{GCI}(t)$ is computationally intensive as it requires estimating quantiles of the pivotal quantity, G(t) (see Equation 11) which is obtained through extensive (e.g., 100 000) [24] Monte Carlo simulations of three $\chi^{2}$ distributions. A less computationally intensive method to obtain ${\hat{v}}_{GCI}(t)$ was suggested by Dobbin and Ionan [18] using two variance reduction techniques [37]. First, a modified version of a variance reduction technique called Rao‐Blackwellization [38] is used, termed the ‘inverse Rao‐Blackwellization estimator’,

(14) $$\begin{array}{ll} & {Ĝ}_{RB}(\alpha )\\ & \;=inf\left\{g:\alpha \leq \frac{1}{M}\sum_{m=1}^{M}P[g(t)\leq g|{\left(\chi_{n-1}^{2}\right)}_{m,}{\left(\chi_{\left(n-1)(k-1\right)}^{2}\right)}_{m}]\right\}, \end{array}$$

where ${\left(\chi_{n-1}^{2}\right)}_{m},{\left(\chi_{\left(n-1)(k-1\right)}^{2}\right)}_{m}$ are $m^{th}$ Monte Carlo observations of $\chi^{2}$ variables to estimate MSS and MSE. Using this estimator, one can obtain the conditional probability in Equation (14) directly from the percentiles of an inverted Gamma‐distribution, thus reducing the computational complexity. Second, a variance reduction technique using control variates [39] was applied [18]. Identifying an approximate linear relationship between v(t)=Ĝ(1−α/2)−Ĝ(α/2) and the control variate $\frac{MSR}{MSS}$ when ρ>0.7, the lower and upper limits of the (1−α)×100% confidence interval are obtained as,

(15)  ÛGCIctrl=ÛGCI−cov(ÛGCI,MSRMSS)var(MSRMSS)×(MSRMSS−E[MSRMSS]),and LˆGCIctrl=LˆGCI−cov(LˆGCI,MSRMSS)var(MSRMSS)×(MSRMSS−E[MSRMSS]),

where ${Û}_{GCI}={Ĝ}_{RB}(1-\alpha /2)$ and ${\hat{L}}_{GCI}={Ĝ}_{RB}(\alpha /2)$ are the upper and lower limits based on the inverse Rao‐Blackwellization estimator, $E[MSR/MSS]=\frac{n\sigma_{r}^{2}+\sigma_{e}^{2}}{k\sigma_{s}^{2}+\sigma_{e}^{2}}\times \frac{n-1}{n-3}$ and $var(MSR/MSS)=\frac{n\sigma_{r}^{2}+\sigma_{e}^{2}}{k\sigma_{s}^{2}+\sigma_{e}^{2}}\times \frac{2{\left(n-1\right)}^{2}(n+k-4)}{\left(k-1){\left(n-3\right)}^{2}(n-5\right)}$. The remaining terms related to the mean squares in Equation (15) are obtained from the Monte Carlo simulation output. The width estimator is then obtained as,

(16) $${\hat{v}}_{GCI}^{ctrl}={Û}_{GCI}^{ctrl}-{\hat{L}}_{GCI}^{ctrl}.$$

When $\omega >\mu_{MLS_{A}}$ or $\omega >\mu_{GCI}$, Dobbin and Ionan [18] proposed an algorithm to obtain the sample size n when the other parameters in q are given. We implemented a slightly modified version of the algorithm (see Figure A1 in Appendix A), where $\hat{v}$ is replaced by ${\hat{v}}_{MLS_{A}}$ in case of $MLS_{A}$, or by ${\hat{v}}_{GCI}$ in case of GCI). The difference in our implemented algorithm and the one by Dobbin and Ionan concerns the steps of the bisection algorithm. Specifically, where Dobbin and Ionan [18] proposed to obtain a sample size by applying a bisection algorithm in several intervals within the range of plausible values of n, we perform the bisection algorithm over the entire region ($n_{min},n_{max}$) in a single step. Both procedures lead to the same solution ($n^{*}$).

The same procedure can be applied by fixing n and considering $g(k)=\omega -{\hat{v}}_{MLS_{A}}(\sigma_{s}^{2},\sigma_{r}^{2},\sigma_{e}^{2},n,k)$. Dobbin and Ionan [18] proposed numerical methods reducing the computational complexity of the $MLS_{A}$ and GCI methods, leading to feasible sample size determination procedures. Note that, unlike the previous procedures, this cannot be easily extended to other confidence interval methods. However, the simulation results showed that, although the width of the confidence interval is quite large, the GCI method provides adequate coverage in almost all scenarios. The $MLS_{A}$ method, however, yields over‐coverage in all scenarios that we studied.

Table 4 shows the summary of the sample sizes obtained by the procedure by Dobbin and Ionan [18] using the expected width of the 95% two‐sided confidence interval for the methods $MLS_{A}$ and GCI. The values for the number of participants for $MLS_{A}$ and GCI indicate the average number of participants obtained using the procedure with 100 replications, while the values in the brackets indicate their standard deviation. The sample size was determined for ρ∈{0.7,0.8,0.9}, R∈{0.1,2}, k∈{5,10} such that the expected width of the confidence interval is less than ω, ω∈{0.2,0.3}. Comparing the sample sizes for R=0.1 to the sample sizes obtained in Table 3 for $VP_{F}$, we see that $VP_{F}$ requires a smaller number of participants for the same ω and k. This is to be expected since the average width of the confidence interval for $VP_{F}$ is smaller than that of $MLS_{A}$ or GCI (see Supporting Information Table S1).

**TABLE 4:** Mean and standard deviation of the number of participants obtained by repeating the sample size determination procedure by Dobbin and Ionan [18] 100 times. The sample size is obtained each time using the expected width of the 95% two‐sided confidence interval for the methods $MLS_{A}$ and GCI, for ρ∈{0.7,0.8,0.9}, R∈{0.1,2}, k∈{5,10}, and ω∈{0.2,0.3} utilizing $10^{3}$ Monte Carlo simulations for GCI and $10^{5}$ for $MLS_{A}$. The values in brackets are the standard deviation (s.d.) computed on the 100 replications. The blanks in the sample size indicate that a width of the confidence interval less than ω could not be obtained with $n\leq 10^{7}$.

			ω=0.2		ω=0.3	
			$MLS_{A}$	GCI	$MLS_{A}$	GCI
R	k	ρ	mean(n),(s.d.(n))	mean(n),(s.d.(n))	mean(n),(s.d.(n))	mean(n),(s.d.(n))
0.1	5	0.7	185.3 (0.95)	96.2 (15.62)	41.1 (0.27)	29.6 (5.03)
		0.8	73.5 (0.50)	46 (7.19)	22 (0)	17.7 (2.73)
		0.9	18.7 (0.47)	15.2 (1.95)	8.3 (0.44)	7.8 (1.06)
	10	0.7	58 (0)	42.4 (7.67)	22 (0)	18.3 (3.22)
		0.8	31 (0)	24.9 (4.28)	13 (0)	11.7 (1.9)
		0.9	11 (0)	10.4 (1.46)	6 (0)	5.7 (0.67)
2	5	0.7				
		0.8				
		0.9			7706.7 (4301.11)	287.9 (46.26)
	10	0.7			861.4 (24.85)	150.7 (20.72)
		0.8			41.9 (0.39)	25.8 (3.39)
		0.9	30 (0.17)	21.2 (2.52)	8 (0)	6.7 (0.74)

Table 4 shows that in some scenarios the sample size determination procedure did not return a finite value, denoted by the blanks in the table. This occurs when the asymptotic width of the confidence interval cannot be achieved for the given R, k, and ρ (see Appendix B for further details). Comparing the scenarios with blanks in Table 4 to the average widths of the confidence interval in Table B2 (Appendix B), it can be seen that the average widths in those scenarios are larger than ω. For example, when ρ=0.7, k=5 and R=2, the asymptotic widths of the confidence interval are 0.46 and 0.42 for $MLS_{A}$ and GCI, respectively, which are larger than 0.3, the maximum ω in Table 4. This implies that when n→∞, the minimum width of the confidence intervals for given ρ, k, and ω is greater than 0.3, and therefore a sample size with ω=0.3 cannot be obtained. In some scenarios however, for example for ρ=0.9, k=5 and R=2, the asymptotic average width of $MLS_{A}$ is 0.298 which is rather close to ω=0.3 and then, a large standard deviation for n was observed in Table 4. We, therefore, recommend that researchers check the asymptotic width of the confidence interval for their choices of ρ, R, and k before proceeding with sample size determination via this procedure.

Furthermore, we also observe blanks (i.e., n>500) for sample sizes determined using $MLS_{G}$ in Table 3, implying that the asymptotic width of $MLS_{G}$ is larger than ω for those scenarios. Comparing the blanks in Tables 3 and 4 for $MLS_{G}$ and $MLS_{A}$, respectively, we notice that a finite sample size (n≤500) could also not be obtained for $MLS_{A}$ for similar combinations of R, k, ρ and ω. Since an expression for the asymptotic width of the confidence interval for $MLS_{G}$ is too complicated, this suggests that one can use the asymptotic width of the confidence interval for $MLS_{A}$ instead.

Table 4 further shows the sample sizes obtained with this procedure, with $MLS_{A}$ generally larger than that with GCI. Furthermore, the standard deviation of the sample sizes obtained by repeating the procedure with both confidence interval methods was quite large, especially when R=2. Therefore, when R>1, to obtain a sample size with a small mean and standard deviation using the procedure by Dobbin and Ionan [18], we recommend choosing a large value for k if possible (e.g., k>10). Furthermore, we recommend that researchers repeat the procedure multiple times to decide on an adequate sample size.

### 5.3 Guide to Choose a Sample Size Determination Procedure

Three procedures are available in the literature for determining the sample size that ensures that the expected width of the confidence interval for ρ remains below a pre‐specified value. These procedures differ in their computational burden and flexibility in selecting confidence interval methods for implementation.

The choice of a confidence interval method directly influences the selection of an appropriate sample size procedure. Table 5 summarizes our recommendations for choosing a confidence interval method and a corresponding sample size procedure. Following our recommendations in Section 4.4, when R≤0.1 and k<8, selecting the $VP_{F}$ method requires using the procedure by Doros and Lew. In other cases, GCI or $MLS_{G}$ can be selected as the confidence interval method. While both methods can be implemented using the procedure by Doros and Lew, its high computational burden makes the procedure by Dobbin and Ionan a more practical choice. However, this limits the choice of the confidence interval to GCI. Note that in this case, the procedure must be repeated several times (e.g., 100 times), and the sample size can be determined based on a summary measure (e.g., the mean) from those repetitions.

**TABLE 5:** Recommendations for selecting a sample size determination procedure and a confidence interval method, based on the combined results of coverage probability, average confidence interval width, and the evaluation of sample size determination procedures.

	R≤0.1	R>0.1
k<8	Purely simulation‐based procedure ($VP_{F}$)	Procedure according to Dobbin and Ionan (GCI)
k≥8	Procedure according to Dobbin and Ionan (GCI)	Procedure according to Dobbin and Ionan (GCI)

### 5.4 Software for Sample Size Calculation

Currently, to the best of our knowledge, there is no available software to obtain sample sizes for the ICC for agreement when the data can be modeled using the two‐way ANOVA model given in Equation (1). Therefore, a shiny app was developed and made available in Github, enabling researchers to obtain sample sizes of participants and/or raters using the procedures described in Section 5. Additionally, the app permits the calculation of the asymptotic width for $MLS_{A}$, GCI and $VP_{F}$, as well as determining confidence intervals for empirical data using the methods $MLS_{A}$, $MLS_{G}$, GCI and $VP_{F}$.

## 6 Empirical Illustration

In this section, we illustrate how the confidence interval methods discussed in Sections 3 and 4, and the sample size determination approaches introduced in Section 5 can be used within the context of a reliability study. The study by Engelhart et al. [40] is used for the purpose of this illustration. This is an obstetric study measuring fetal heart rate (FHR). During intermittent auscultation, the heartbeat of the fetus is measured by midwives using a hand‐held Doppler device. If an abnormality (acceleration or deceleration from the baseline heart rate) is detected, then a treatment decision is made. Inter‐rater agreement and reliability are important in this context as disagreements may lead to different interventions for the mother and her baby. The primary aim of the study by Engelhart et al. [40] was to evaluate the inter‐rater reliability and agreement of fetal heart rate measured using a handheld Doppler device (beats per minute, bpm). The FHR was determined on the same n=154 women undergoing labor by k=16 midwives. After removing missing values, data from 145 FHRs by 16 midwives remained.

The data from this study can be suitably modeled using the two‐way ANOVA model (see Equation 1). The ANOVA model makes the assumptions of normality of the FHR measurements by each midwife and that the variance of the measurements by different midwives is homoscedastic. Exploratory analysis revealed that the absolute value of the skewness is less than 1 and the value of the excess kurtosis (averaged across midwives) is less than 2, implying that the midwife measurements can be considered normal [41, 42]. Mild heteroscedasticity is observed. The estimated ICC for agreement, $\hat{\rho }=0.735$, and the estimated ratio of rater‐to‐error variance, $\hat{R}=0.09$. The value of the ICC for agreement indicates that 73.5% of the FHR variability can be attributed to variability between the fetuses. The confidence interval for the ICC for agreement obtained using the seven confidence interval methods is given in Table 6. The estimated ICC for agreement, $\hat{\rho }=0.735\;(0.682,0.781)$, was considered to be sufficient for using the measurements in FHR monitoring.

**TABLE 6:** Confidence intervals for the ICC for agreement using the seven confidence interval methods.

Limit	$W_{log}$	$W_{mat}$	$MLS_{A}$	$MLS_{G}$	GCI	$VP_{F}$	$VP_{B}$
Lower	0.688	0.683	0.672	0.684	0.682	0.681	0.685
Upper	0.786	0.787	0.786	0.783	0.781	0.779	0.782

From Table 6 it can be seen that the limits of the confidence intervals are rather close across the different methods. The width of the confidence interval for the different methods is approximately 0.1. Considering that the value for the ICC for agreement for the population of fetuses and midwives is around ρ=0.7 and the corresponding rater‐to‐error variance ratio is R≤0.1, from Figure 1, we can deduce that all the confidence interval methods provide adequate coverage for n=145 and k=16.

We now illustrate how the different sample size determination procedures can be used to plan a reliability study. A researcher may be interested in planning a reliability study to measure the FHR (bpm) using a handheld Doppler device. Suppose that the researchers expect the ICC for agreement to be 0.7, and want to obtain sample sizes such that the width of the confidence interval is less than 0.1. The researchers further assume the rater‐to‐error variance ratio to be 0.1 or less. Then, if the number of raters should be between 10 and 15, following our recommendations in Section 4.4 and Section 5.3, the researchers can obtain sample sizes using the procedure of Dobbin and Ionan [18] described in Section 5.2 using the confidence interval method GCI. The procedure is repeated 100 times and the mean (and standard deviation) of the calculated number of participants indicates that the researchers can opt for 225 (±41) participants when a maximum of 10 raters are available, or 167 (±31) participants when a maximum of 15 raters are available for the reliability study.

## 7 Discussion

During the planning stage of a reliability study, it is essential to determine the number of participants and/or raters to limit the uncertainty associated with the sampling process. When the measurements from the reliability study can be modeled using a two‐way ANOVA model as in Equation (1), two different ICC coefficients can be obtained, namely the ICC for agreement and the ICC for consistency. In this paper, we provide an overview of the different procedures that were developed to determine the number of participants and/or the number of raters for a reliability study when using the ICC for agreement. These procedures are based on the expected width of the confidence interval.

Before examining sample size determination procedures, we evaluated the statistical properties of seven confidence interval methods for the ICC for agreement, completing the comparison of all available methods. Our simulation study highlighted the critical role of the rater‐to‐error variance ratio, R, in determining whether a method achieves coverage probabilities close to the nominal level. The results showed that GCI consistently provides the closest coverage probability to the nominal level across scenarios. $MLS_{G}$ performed similarly to GCI with respect to its coverage probability under most scenarios, with comparable interval widths. These findings align with Ionan et al. [11] earlier study. The estimator of the ICC is a ratio with both numerator and denominator as linear combinations of scaled $\chi^{2}$ random variables. GCI does not rely on approximations but leverages a large number of simulations utilizing samples from scaled $\chi^{2}$‐distributions (see Equation 11). $MLS_{G}$ also avoids standard approximation techniques, having its lower and upper limits derived separately using the Fieller‐type confidence interval for ratios [43]. As a result, GCI and $MLS_{G}$ are expected to perform well. However, note that, when k≤8, the confidence intervals according to $MLS_{G}$ and GCI are relatively wide. $VP_{F}$, a method not included in prior comparisons, performed well for R≤0.1, offering narrower confidence intervals while maintaining a coverage probability that was the closest to the nominal level. For k≥8, GCI and $MLS_{G}$ intervals approach the width of $VP_{F}$. Higher values of ρ (e.g., ρ=0.8, 0.9; see Supporting Information Table S1) improved coverage probabilities and provided shorter interval widths. Based on these results, we recommend using GCI or $MLS_{G}$ for k≥8. For k<8, $VP_{F}$ is preferable if R≤0.1; otherwise, $MLS_{G}$ or GCI is recommended.

Three procedures to determine the optimal combinations of n and k for a reliability study were identified in the literature. These procedures aim to identify the combination of n and k by exploring a grid of their feasible values, such that the width of the confidence interval is less than a pre‐defined constant ω when a planning value for ρ and R is given. The first procedure, proposed by Saito et al. [7] uses an analytical formula to search within a small grid of feasible n and k values. However, this method requires specifying a range for the total number of observations N=n×k, which may result in combinations of n and k that are not feasible in practice. Additionally, if the specified range of N is too broad (e.g., the range of N exceeds three or four values), the procedure can become rather time‐intensive. For these reasons, this procedure was not investigated further. The second procedure, proposed by Doros and Lew [17], involves exploring a grid of all feasible values of n and k and uses simulations to calculate the expected confidence interval width for each combination. Although general in its applicability, this approach is also computationally intensive. We adapted this procedure by fixing k and exploring a grid of plausible n, making it computationally less intensive. The third procedure, introduced by Dobbin and Ionan [18] is based on analytical formulas for the confidence interval widths of $MLS_{A}$ and GCI. While computationally the most efficient, this procedure is limited to these two confidence interval methods and may not be easily adapted to others. Further, it was observed that for some combinations of ρ and R, no finite sample sizes could be obtained for a given value of k. In such cases, the asymptotic width of the confidence interval methods $MLS_{A}$ and GCI derived by Dobbin and Ionan [18] allows to calculate, via simulation, the minimum width the confidence interval one can achieve under the given scenarios. We recommend that researchers examine the asymptotic width of the confidence interval for their chosen ρ, R, and k before proceeding to sample size determination. Furthermore, repeating the sample size procedure by Dobbin and Ionan [18] multiple times, we found that the resulting sample sizes could be rather different. Therefore, we urge researchers using this procedure to repeat the procedure several times and obtain the final sample size using some summary measure, such as taking the mean of sample sizes obtained by repeating this procedure 100 times.

Our findings demonstrate that the choice of sample size determination procedure and confidence interval method significantly impacts the final combination of raters, k, and participants, n, with results highly dependent on the planning values for ρ and R. We therefore advise researchers to carefully consider the requirements of the reliability study as a guide to choose the appropriate procedure. As for the choice of the values of ρ and R to determine a sample size, when one is uncertain about their values, we recommend choosing ρ as small as plausible and R as large as plausible, since this would lead to a conservative sample size. As for the choice of the sample size determination procedure, one may use the procedure of Doros and Lew [17] (adapted by fixing k) for $MLS_{G}$ and $VP_{F}$, or of Dobbin and Ionan [18] for $MLS_{A}$ and GCI. The use of these procedures was illustrated using FHR measurements by Engelhart et al. [40]. In addition, these procedures were all implemented in an R Shiny app, which enables users to obtain sample sizes via an interactive and easy‐to‐use graphical interface.

Our work improves the understanding of the methods to construct a confidence interval for the ICC for agreement by making a comparison of all available methods. Additionally, we evaluate procedures for determining sample sizes for reliability studies, ensuring a precision around the ICC estimate, and offer tools and recommendations for designing a reliability study. However, our study is not without limitations. First, the estimator for the ICC for agreement and its corresponding confidence interval rely on the assumptions of normality of the outcomes as well as homoscedasticity of the variances, in line with the two‐way ANOVA model mentioned in Equation 1. The statistical properties of these confidence intervals are impacted by violations of these conditions. We therefore advise researchers to carefully check the assumptions of the ANOVA model before applying the methods mentioned in this paper. Ionan et al. [11] reported that the methods showing good coverages, that is, GCI and $MLS_{G}$, are not robust to violations of normality. In such a case, one may use the non‐parametric estimation of the ICC introduced by Lu et al. [44], which does not make the assumption of normality. However, a procedure for sample size determination is not yet available in this case. Second, in this study, we consider a complete design, that is, each rater rates every participant. This may not be the case when, for example, some measurements are missing by design. The estimation of the ICC for agreement in such a case has been discussed by ten Hove [45], however, no sample size determination has yet been developed under this scenario. Third, the ANOVA model considered in this paper does not include an interaction term. However, when raters rate each participant more than once, an interaction effect between the raters and participants can be included in the model and estimated from the data. Whether the same methods discussed in this paper can be extended to such a design requires further investigation. Fourth, in this paper, we reviewed the sample size determination procedures under the width of the confidence interval approach. In another approach, researchers may be interested in finding sample sizes that ensure a high probability of the width of the confidence interval being less than a pre‐specified value. This is known as the assurance probability approach [3, 46]. How to extend the methods studied in this manuscript, analytically and/or computationally, to accommodate the assurance probability approach is left for future work. Fifth, a researcher may, however, be interested in determining sample sizes for a study where the aim is to statistically test whether the ICC for agreement is larger than a pre‐defined value. No work has yet been done in this regard, and hence it is a topic for future research.

## Conflicts of Interest

The authors declare no conflicts of interest.

## Supporting information


**Data S1**. Appendices.

**Table S1**. Coverage of the seven confidence interval methods for different combinations of n, k, R and rho. Average width of the seven confidence interval methods for different combinations of n, k, R and rho.

## Data Availability

The data that supports the findings of this study are available in the Supporting Information of this article.

## Appendix B Asymptotics

### Asymptotics for the Generalized Confidence Interval

#### n→∞

Denoting $k_{lim}^{2}=\lim_{n\to \infty }\frac{1}{k-1}\sum_{j=1}^{k-1}{\left({\bar{y}}_{.j}-{\bar{y}}_{..}\right)}^{2}$, under the assumptions of the ANOVA model (Equation 1) the pivotal quantity reduces to [18],

where  represent convergence in distribution and $W_{1}∼\chi_{k-1}^{2}$.

#### k→∞

Denoting $n_{lim}^{2}=\lim_{k\to \infty }\frac{1}{n-1}\sum_{i=1}^{k-1}{\left({\bar{y}}_{i.}-{\bar{y}}_{..}\right)}^{2}$, under the assumptions of the ANOVA model Equation (1) [18], the pivotal quantity reduces to,

where $W_{2}∼\chi_{n-1}^{2}$.

### Asymptotics for the Modified Large Sample Confidence Interval

The derivation of the asymptotics by Dobbin and Ionan [18] utilizes the expression of the limits of the confidence interval method $MLS_{A}$.

#### n→∞

Denoting $k_{lim}^{2}=\lim_{n\to \infty }\frac{1}{k-1}\sum_{j=1}^{k}{\left({\bar{y}}_{.j}-{\bar{y}}_{..}\right)}^{2}$, the lower(L) and upper(U) limits of the confidence interval can be written as [18],

$$\begin{array}{ll} & L\overset{d}{\to }max\{0,\frac{\sigma_{s}^{2}}{\sigma_{s}^{2}+\sigma_{e}^{2}+k_{lim}^{2}/F_{k-1,\infty }(\frac{\alpha }{2})}\},\;\text{and}\\ & U\overset{d}{\to }max\{0,\frac{\sigma_{s}^{2}}{\sigma_{s}^{2}+\sigma_{e}^{2}+k_{lim}^{2}/F_{k-1,\infty }(1-\frac{\alpha }{2})}\} \end{array}$$

#### k→∞

Denoting $n_{lim}^{2}=\lim_{k\to \infty }\frac{1}{n-1}\sum_{i=1}^{n}{\left({\bar{y}}_{i.}-{\bar{y}}_{..}\right)}^{2}$, the lower(L) and upper(U) limits of the confidence interval can be written as [18],

$$\begin{array}{ll} & L\overset{d}{\to }max\{0,\frac{F_{\infty ,n-1}(\frac{\alpha }{2})n_{lim}^{2}}{\sigma_{r}^{2}+\sigma_{e}^{2}+F_{\infty ,n-1}(\frac{\alpha }{2})n_{lim}^{2}}\},\;\text{and}\\ & U\overset{d}{\to }max\{0,\frac{F_{\infty ,n-1}(1-\frac{\alpha }{2})n_{lim}^{2}}{\sigma_{r}^{2}+\sigma_{e}^{2}+F_{\infty ,n-1}(1-\frac{\alpha }{2})n_{lim}^{2}}\} \end{array}$$

### Asymptotics for the Variance Partitioning Confidence Interval

We derive the asymptotic expression for the variance partitioning confidence interval ($VP_{F}$) considering the case where the estimator of the variance components and the variance of the variance components are constructed via ANOVA estimation. Recall that the distribution of $\hat{\rho }$ when using the confidence interval method $VP_{F}$, is given by (see Section 3.4.1),

$$\begin{array}{l} \frac{{\hat{\sigma }}_{s}^{2}f_{{\hat{df}}_{1}{\hat{df}}_{2}}}{{\hat{\sigma }}_{s}^{2}f_{{\hat{df}}_{1}{\hat{df}}_{2}}+{\hat{\sigma }}_{E}^{2}} \end{array}$$

where ${\hat{df}}_{1}=\frac{2{\hat{\sigma }}_{s}^{4}}{\hat{V}({\hat{\sigma }}_{s}^{2})}$ and ${\hat{df}}_{2}=\frac{2{\hat{\sigma }}_{E}^{4}}{\hat{V}({\hat{\sigma }}_{E}^{2})}=\frac{2{\left({\hat{\sigma }}_{e}^{2}+{\hat{\sigma }}_{r}^{2}\right)}^{2}}{\hat{V}({\hat{\sigma }}_{e}^{2})+\hat{V}({\hat{\sigma }}_{r}^{2})}$, respectively. To find the expression for the asymptotic width of the confidence interval, we need the asymptotic expressions for ${\hat{\sigma }}_{s}^{2}$, ${\hat{\sigma }}_{r}^{2}$, ${\hat{\sigma }}_{e}^{2}$, $\hat{V}({\hat{\sigma }}_{s}^{2})$, $\hat{V}({\hat{\sigma }}_{r}^{2})$ and $\hat{V}({\hat{\sigma }}_{e}^{2})$.

Following Dobbin and Ionan [18], ${\hat{\sigma }}_{s}^{2}$ converges in distribution to $\sigma_{s}^{2}$, and ${\hat{\sigma }}_{e}^{2}$ converges in distribution to $\sigma_{e}^{2}$, and ${\hat{\sigma }}_{r}^{2}$ converges in distribution to $k_{lim}^{2}=\lim_{n\to \infty }\frac{1}{k-1}\sum_{j=1}^{k}{\left({\bar{y}}_{.j}-{\bar{y}}_{..}\right)}^{2}$ (see Appendix B of Dobbin and Ionan [18]).

We obtain the variance of the estimator of the variances from the variance of mean squares given in Section 3.1.1. The estimated variance of the estimator of the variance of the participants is obtained as,

$$\begin{array}{ll} \hat{V}({\hat{\sigma }}_{s}^{2}) & =\frac{1}{k^{2}}(V(MSS)+V(MSE))\\ & =\frac{2}{k^{2}(n-1)}\left({\left(k{\hat{\sigma }}_{s}^{2}+{\hat{\sigma }}_{e}^{2}\right)}^{2}+\frac{{\left({\hat{\sigma }}_{e}^{2}\right)}^{2}}{k-1}\right). \end{array}$$

This converges in distribution to,

$$\begin{array}{l} V({\hat{\sigma }}_{s}^{2})=\frac{2}{k^{2}(n-1)}({\left(k\sigma_{s}^{2}+\sigma_{e}^{2}\right)}^{2}+\frac{\sigma_{e}^{4}}{k-1}). \end{array}$$

Therefore, under the limit n→∞, $V({\hat{\sigma }}_{s}^{2})\to 0$.

The estimated variance of the estimator of the variance of the raters is obtained as,

$$\begin{array}{ll} \hat{V}({\hat{\sigma }}_{r}^{2}) & =\frac{1}{n^{2}}(V(MSR)+V(MSE))\\ & =\frac{2}{n^{2}(k-1)}\left({\left(n{\hat{\sigma }}_{r}^{2}+{\hat{\sigma }}_{e}^{2}\right)}^{2}+\frac{{\left({\hat{\sigma }}_{e}^{2}\right)}^{2}}{n-1}\right)\\ & =\frac{2}{\left(k-1\right)}\left({\left({\hat{\sigma }}_{r}^{2}+\frac{{\hat{\sigma }}_{e}^{2}}{n}\right)}^{2}+\frac{{\left({\hat{\sigma }}_{e}^{2}\right)}^{2}}{n^{2}(n-1)}\right). \end{array}$$

This converges in distribution to,

$$\begin{array}{l} V({\hat{\sigma }}_{r}^{2})=\frac{2}{\left(k-1\right)}\left({\left(k_{lim}^{2}+\frac{\sigma_{e}^{2}}{n}\right)}^{2}+\frac{\sigma_{e}^{4}}{n^{2}(n-1)}\right). \end{array}$$

Therefore, under the limit n→∞, $V({\hat{\sigma }}_{r}^{2})\to \frac{2{\left(k_{lim}^{2}\right)}^{2}}{k-1}$.

The estimated variance of the estimator of the variance of the errors is obtained as,

$$\begin{array}{l} \hat{V}({\hat{\sigma }}_{e}^{2})=V(MSE)=\frac{2{\left({\hat{\sigma }}_{e}^{2}\right)}^{2}}{\left(n-1)(k-1\right)}. \end{array}$$

This converges in distribution to,

$$\begin{array}{l} V({\hat{\sigma }}_{e}^{2})=\frac{2\sigma_{e}^{4}}{\left(n-1)(k-1\right)}. \end{array}$$

Therefore, under the limit n→∞, $V({\hat{\sigma }}_{e}^{2})\to 0$.

Then, plugging $V({\hat{\sigma }}_{s}^{2})$ in the equation for ${\hat{df}}_{1}$ gives, under the limit n→∞, ${\hat{df}}_{1}\to \infty$. Plugging $V({\hat{\sigma }}_{r}^{2})$ and $V({\hat{\sigma }}_{e}^{2})$ in the equation for ${\hat{df}}_{2}$ gives, under the limit n→∞, ${\hat{df}}_{2}\to (k-1){\left(1+\frac{\sigma_{e}^{2}}{k_{lim}^{2}}\right)}^{2}$.

Using Slutsky's theorem, the limits of the confidence interval can now be written as,

When R is small however, for example R≤0.1, numerical results presented in Table B1 show that $k_{lim}^{2}$ can be replaced by $\sigma_{r}^{2}$. Note that, in Section 3.4, the variance of the variance components is based on maximum‐likelihood estimation. However, when n→∞ and k→∞, ANOVA and ML estimation lead to similar results (see Searle et al. [25]).

**TABLE B1:** Comparison of the Asymptotic Width of the Confidence Interval With $VP_{F}$ Under Different Scenarios, Using Different Specifications of $df_{2}$, That Is, $df_{2}=(k-1){\left(1+\frac{\sigma_{e}^{2}}{k_{lim}^{2}}\right)}^{2}$ (Model1), and $df_{2}=(k-1){\left(1+\frac{\sigma_{e}^{2}}{\sigma_{r}^{2}}\right)}^{2}$ (Model2). the Results for Models 1 and 2 Are Obtained Using n=1000 and Using $10^{5}$ Simulations.

ρ	k	R	Model1	Model2
0.7	2	0.1	0.0942	0.1053
0.8	2	0.1	0.0735	0.0802
0.9	2	0.1	0.0429	0.0452
0.7	5	0.1	0.0517	0.0528
0.8	5	0.1	0.0397	0.0403
0.9	5	0.1	0.0225	0.0227
0.7	10	0.1	0.0352	0.0353
0.8	10	0.1	0.0269	0.0269
0.9	10	0.1	0.0152	0.0151

### Results of the Asymptotic Width With the Generalized Confidence Interval, the Modified Large Sample Confidence Interval, and Variance Partitioning Method

In Table B2 the asymptotic width of the confidence interval with $MLS_{A}$ and GCI are obtained using simulations, according to Dobbin and Ionan [18] (see Appendix B). As for the width of the confidence interval with $VP_{F}$, the asymptotic width of the confidence interval is obtained analytically following our derivation (see Model2 in Appendix B).

**TABLE B2:** Width of the Confidence Interval With $MLS_{A}$, GCI, and $VP_{F}$ for k∈{5,10}, R∈{0.1,2}, and ρ∈{0.7,0.8,0.9}. the Results for $MLS_{A}$ and GCI Are Obtained Using n=1000, and the Table Shows the Average Width of the Confidence Interval From $10^{5}$ Simulations, While the Values in Brackets Are the Standard Deviation of the Widths. the Results for $VP_{F}$ Are Obtained Analytically (According to Model2).

			$MLS_{A}$	GCI	$VP_{F}$
k	R	ρ	E[W],sd(W)	E[W],sd(W)	E[W]
5	0.1	0.7	0.120 (0.065)	0.100 (0.061)	0.053
		0.8	0.098 (0.057)	0.080 (0.052)	0.040
		0.9	0.059 (0.038)	0.048 (0.034)	0.023
	2	0.7	0.458 (0.113)	0.421 (0.127)	
		0.8	0.412 (0.131)	0.369 (0.139)	
		0.9	0.298 (0.128)	0.256 (0.126)	
10	0.1	0.7	0.051 (0.021)	0.047 (0.021)	0.035
		0.8	0.040 (0.017)	0.036 (0.017)	0.027
		0.9	0.023 (0.01)	0.021 (0.01)	0.015
	2	0.7	0.287 (0.063)	0.272 (0.067)	
		0.8	0.237 (0.066)	0.221 (0.068)	
		0.9	0.148 (0.053)	0.136 (0.052)	

## Appendix C Revisions to Published Formulas

### Modified Large Sample Confidence Interval for ρ ($MLS_{A}$)

The derivation of the upper and lower limits of the confidence interval for $MLS_{A}$ does not correspond to the final equation for the limits of the confidence interval for the transformation of ρ. The final expression provided in Arteaga et al. [9] (Equation 2.9) is,

The correct expression should be

$$\begin{array}{ll} L_{A}^{*} & =\frac{-1+F_{\alpha /2:n_{1},\infty }^{-1}F_{1}+(1-F_{\alpha /2:n_{1},\infty }^{-1}F_{\alpha /2:n_{1},n_{3}})F_{\alpha /2:n_{1},n_{3}}F_{1}^{-1}}{n_{1}+F_{\alpha /2:n_{1},\infty }^{-1}F_{\alpha /2:n_{1},n_{2}}F_{2}},\\ U_{A}^{*} & =\frac{\begin{array}{c} -\;1+F_{1-\alpha /2:n_{1},\infty }^{-1}F_{1}+(1-F_{1-\alpha /2:n_{1},\infty }^{-1}F_{1-\alpha /2:n_{1},n_{3}})F_{1-\alpha /2:n_{1},n_{3}}F_{1}^{-1} \end{array}}{n_{1}+F_{1-\alpha /2:n_{1},\infty }^{-1}F_{1-\alpha /2:n_{1},n_{2}}F_{2}}. \end{array}$$

For a definition of all quantities in the above equation, consult Arteaga et al. [9].

### Generalized Confidence Interval Method

The expression for the generalized pivotal quantity given by Ionan et al. [11](in Appendix A, Page 10) contains a typo for $c_{4}$. The expression is

where $c_{2}=(b_{0}-1)/(l_{0}r_{0})$, $c_{3}=(b_{0}l_{0}r_{0}-b_{0}-l_{0}+1)/(l_{0}r_{0})$, $c_{1}=(l_{0}-1)/(b_{0}r_{0})$, and $c_{4}=c_{3}(b_{0}l_{0}r_{0}-b_{0}-l_{0}+1)/b_{0}$.

The correct expression for $c_{4}$ should be $c_{4}=c_{3}(b_{0}l_{0}r_{0}-b_{0}-l_{0})/b_{0}$. For a definition of all quantities in the above equation, consult Ionan et al. [11].
