# Supplementary material for: Confidence Intervals and Sample Size for the ICC in Two‐Way ANOVA Models
Source: Stat Med. 2025 May 22;44(10-12):e70106. doi: 10.1002/sim.70106 (PMC12099031; doi:10.1002/sim.70106)
Supplement: Supplementary file 1 — Data S1. Appendices. [file SIM-44-0-s001.pdf]

Supplementary File 1

A | OTHER CONFIDENCE INTERVAL METHODS AVAILABLE IN THE LITERATURE

A.1 | Moment approximation method

Under the moment approximation method introduced by Fleiss and Shrout[1], the lower and upper bounds of the $(1-\alpha)\times 100\%$ confidence interval for ρ are[2],

$$L = \frac{n(MSS - F_{\nu,(n-1)}(1-\alpha/2)MSE)}{F_{\nu,(n-1)}(1-\alpha/2)\{kMSR + (kn-k-n)MSE\} + nMSS},$$
$$U = \frac{n(F_{(n-1),\nu}(1-\alpha/2)MSS - MSE)}{kMSR + (kn-k-n)MSE + nF_{(n-1),\nu}(1-\alpha/2)MSS}, \quad (1)$$

where $F_{\nu_1,\nu_2}(q)$ denotes the $q\times 100$ percentile of an F-distribution with ν_1 and ν_2 degrees of freedom in the numerator and denominator, respectively. Since an exact sampling distribution of $\hat{\rho}$ (Equation 4) is "too complicated for practical use"[3], the degrees of freedom, ν in Equation 1, are based on approximations. These approximations are moment-based and the confidence interval differs depending on which moments are used in the approximation, which can be two- (ν_S), three- (ν_T), four- (ν_F) moments, or, a combination of the moment approximations (ν_{T^*}, ν_{F^*}). These are briefly described below.

A.1.1 | Two-moments approximation

Fleiss and Shrout[1] applied Satterthwaite's two-moments approximation, thus the degrees of freedom, ν are approximated by ν_S, as,

$$\nu_S = \frac{(a_1 u + a_2)^2}{\frac{a_1^2 u^2}{k-1} + \frac{a_2^2}{(n-1)(k-1)}},$$

where $u = \frac{MSR}{MSE}$, $a_1 = \frac{k\hat{\rho}}{n(1-\hat{\rho})}$ and $a_2 = 1 + \frac{k\hat{\rho}(n-1)}{n(1-\hat{\rho})}$.

A.1.2 | Higher-moment and hybrid approximation

Zou and McDermott[4] attempted to improve the approximate degrees of freedom by including higher moments, under the rationale that using higher moments leads to a better approximation. This led to the three-moment approximation ν_T,

$$\nu_T = \frac{\left[\frac{a_1^2 u^2}{k-1} + \frac{a_2^2}{(n-1)(k-1)}\right]^3}{\left[\frac{a_1^3 u^3}{(k-1)^2} + \frac{a_2^3}{(n-1)^2(k-1)^2}\right]^2},$$

and the four-moment approximation ν_F,

$$\nu_F = \left[\left(\frac{a_1^2u^2}{k-1} + \frac{a_2^2}{(n-1)(k-1)}\right)^3\right] \Big/ \left[\frac{a_1^6u^6}{(k-1)^4}(9(k-1)+52) + \frac{9a_1^4a_2^2u^4}{(n-1)(k-1)^4}(3(k-1)+4) + \frac{32a_1^3a_2^3u^3}{(n-1)^2(k-1)^4} + \frac{9a_1^2a_2^4u^2}{(n-1)^3(k-1)^4}(3(n-1)(k-1)+4) + \frac{a_2^6}{(n-1)^4(k-1)^4}(9(n-1)(k-1)+52)-9\right],$$

where u, a_1 and a_2 are defined as in the two moments approximation. Simulation results[4] showed that coverage probabilities with the two-moment approximation showed under-coverage, that is, the coverage probability of the confidence interval is less than the nominal value, whereas the three- and four-moment approximations showed over-coverage. Therefore, Zou and McDermott[4] further used a combination of the two-moments approximation, with the three-, or four-moments approximations to obtain hybrid degrees of freedom,

$$\nu_{T^*} = w\nu_S + (1-w)\nu_T, \text{ and,}$$
$$\nu_{F^*} = w\nu_S + (1-w)\nu_F,$$

where w $(0 < w < 1)$ is a weight on ν_S, which can be chosen arbitrarily.

A.2 | Modified profile likelihood method

Xiao and Liu[5] introduced a likelihood-based method to obtain the confidence interval for ρ. Denoting $\phi = \frac{\sigma_r^2}{\sigma_s^2+\sigma_r^2+\sigma_e^2}$, and $R = \frac{\sigma_r^2}{\sigma_e^2}$, the profile log-likelihood function is given as[5],

$$l_p(\rho; y_{ij}) = max\{l(\rho, \phi; y_{ij}) : 0 < \phi < 1-\rho\},$$

where,

$$l(\rho, \phi; y_{ij}) = -\frac{1}{2}\Big\{c' + \ln(1+(k-1)\rho+(n-1)\phi) + (n-1)\ln(1+(k-1)\rho-\phi) + (k-1)\ln(1-\rho+(n-1)\phi) + (n-1)(k-1)\ln(1-\rho-\phi) + nk\ \ln\left[\frac{(n-1)MSS}{1-\phi+(k-1)\rho} + \frac{(k-1)MSR}{1+(n-1)\phi-\rho} + \frac{(n-1)(k-1)MSE}{1-\rho-\phi}\right]\Big\}$$

is the log-likelihood function with parameters ρ and ϕ, and c' is a constant. If $\hat{\rho}$ is the maximum likelihood estimate of ρ, the limits of the $(1-\alpha) \times 100\%$ confidence interval for ρ are obtained by solving,

$$\{\rho : 2l_p(\hat{\rho}; y_{ij}) - 2l_p(\rho; y_{ij}) \le (1+\kappa)\chi_1^2(1-\alpha)\}, \tag{2}$$

where κ is a chosen constant and $\chi_1^2(1-\alpha)$ is the $(1-\alpha) \times 100$ percentile of a χ^2-distribution with one degree of freedom. The choice of κ has an impact on the coverage probabilities of the confidence interval. The theoretical value of κ for which the confidence interval produces the expected coverage is denoted by κ_{corr}, for which a closed-form expression is not available. Choosing κ smaller than κ_{corr} leads to under-coverage. On the other hand, setting κ larger than κ_{corr} leads to over-coverage. Xiao and Liu[5] suggested a numerical method to yield an estimate of κ such that the coverage probability of the confidence interval given n, k, R and ρ, is at least equal to or greater than the expected coverage probability. The numerical method involves a two-step process. First, specify a grid of values in the range of plausible values for κ (e.g., –0.8 to 2.0 in steps of 0.05)[5]. Then, for each value of κ in the grid, a large number of simulations (say, $10,000$) are performed, and for each simulation, a root-finding algorithm is used to obtain a confidence interval following Equation 2. An empirical coverage probability is determined as the proportion of confidence intervals from the simulations that contain the true value of ρ. The smallest value of κ for which the coverage probability meets or exceeds the expected coverage probability is selected as the approximation of κ_{corr} obtained through this procedure[5]. This numerical approach thus provides κ_{corr} for fixed values of R and ρ. However, since R and ρ are unknown, a grid spanning their plausible ranges is specified. For each combination of R and ρ within this grid, κ_{corr} is calculated

using the numerical method, resulting in a set of κ_{corr} values corresponding to all grid combinations. Finally, to guarantee an empirical coverage probability equal to or greater than the expected coverage probability, the maximum value of κ_{corr} for the different combinations of ρ and R, is selected as,

$$\kappa_m = max\{\kappa_{corr}(\rho, R) : \rho_L \leq \rho \leq \rho_U; R_L \leq R \leq R_U\}, \tag{3}$$

where ρ_L and ρ_U are the plausible lower and upper limits for ρ, and R_L and R_U for R. An advantage of this method is that it produces a small width of the confidence interval[5] with the confidence interval producing no less than the expected coverage probability. However, this comes at a huge computational cost since this method uses two grid searches. One grid search is for ρ and R, and another is for κ_{corr} within each combination of ρ and R. These grid searches are accompanied by Monte Carlo simulations and a root finding algorithm to obtain a confidence interval for each simulation, making this method computationally expensive and therefore, not really feasible in practice. Further, Equation 3 assumes known limits of ρ and R which is generally not the case.

B | SAMPLE SIZE DETERMINATION PROCEDURE ACCORDING TO SAITO ET AL.[6].

The sample size determination procedure proposed by Saito et al.[6] utilizes the Wald confidence interval to guide the process of defining the grid values for n and k. Saito et al.[6] proposed to consider the Wald confidence interval for ρ on the log-transformed scale. The relevant variance, $V(log(\hat{\rho}))$ (see Equation 6) can then be re-written as a function of the number of raters k, $h(k)$, which can be expressed as,

$$h(k) = 2\frac{\delta k^2 + \epsilon k + \gamma}{k^2 - (N+1)k + N} \tag{4}$$

where,

$$\begin{aligned}
\delta &= -\phi^2 - \frac{2(N-1)}{N}\frac{\phi^2}{R} - \frac{N-1}{N}\frac{\phi^2}{R^2}, \\
\epsilon &= 2\phi^2 - \frac{2}{\rho}\frac{\phi^3}{R} - \frac{2(2N-1)}{N\rho}\frac{\phi^3}{R^2} - \frac{2(N-1)}{N\rho}\frac{\phi^3}{R^3}, \text{ and,} \\
\gamma &= -N\phi^2 + \frac{2}{\rho}\frac{\phi^3}{R} + \frac{2}{\rho}\frac{\phi^3}{R^2} - \left(\frac{1}{\rho}\right)^2\frac{\phi^4}{R^2} - 2\left(\frac{1}{\rho}\right)^2\frac{\phi^4}{R^3} - \left(\frac{1}{\rho}\right)^2\frac{\phi^4}{R^4}, \\
\phi &= \frac{\sigma_r^2}{\sigma_s^2 + \sigma_r^2 + \sigma_e^2}.
\end{aligned}$$

For a fixed $N = n \times k$ one can derive the number of raters, k, that minimizes $V(log(\hat{\rho}))$. Taking the derivate of $h(k)$ in Equation 4 with respect to k, $h'(k)$, and solving $h'(k) = 0$ for k, yields

$$k = \frac{NA + B}{A + B} - \frac{\sqrt{(NA+B)^2 - (A+B)(N^2A + B)}}{A + B}, \tag{5}$$

where, $A = \frac{-\delta-\epsilon-\gamma}{N-1}$ and $B = \frac{n^2k^2\delta + N\epsilon + \gamma}{N-1}$. For a given $N = n \times k$, and for a pre-specified planning value of ρ and R, one can obtain the combination of k (from Equation 5) and n which produces the minimum variance. As it can be seen from Equations 4 and 5, the value of R plays an important role in determining the combination of n and k for a fixed N that minimizes the variance, $V(log(\hat{\rho}))$. When $R \to \infty$, and N is sufficiently large ($\gtrsim$ 50), k can be approximated by,

$$k \simeq \sqrt{N}\left(1 - \frac{1}{R}\right) = \sqrt{N} + \mathcal{O}\left(\frac{1}{R}\right),$$

implying that when the total number of observations is large, the minimum variance is obtained when $k \approx n$. Numerical evaluation of Equation 5 for $R = 1e{-}6$ for $n \times k \in \{4, 10^5\}$ (in steps of 2) and $\rho \in \{0.01, 0.99\}$ (in steps of 0.01) showed that when $\rho \geq 0.5$, the largest k that was obtained from the equation is approximately 2 and when $\rho < 0.5$, the largest k obtained was $k \approx \frac{1}{\rho}$.

It is worth noting that Equation 6 and, thus, Equation 4 rely on a normal approximation. Therefore, the combination of n and k obtained from Equation 4 is used as a starting value to look for the optimal combination of n and k. The expected width

of the confidence interval is obtained from simulations using any of the confidence interval methods described in Section 3. Furthermore, the expected width of the confidence interval for the specified $N = n \times k$ might not be smaller than ω. Therefore, different values of N are used to find the optimal combination of n and k satisfying the sample size criterion.

Saito et al.[6] proposed the following steps in order to calculate the combination of n and k such that the expected width of the confidence interval for ρ is less than a predetermined value, ω.

1. Since the width of the confidence interval depends on R and ρ, possible values of R and ρ need to be provided. When not enough information to select a value of R or ρ is available, a large R and/or a small ρ represent a conservative choice.
2. Start with an arbitrary value of $N = n \times k$. For a fixed N, following Equation 5 one can obtain the values of k and n which minimize the variance of $log(\hat{\rho})$. The calculated k and n may not be integers. As integer values for n and k are required, the width of the confidence interval is obtained in the vicinity of those calculated n and k. This is accomplished by taking the value of k obtained from Equation 5 as the starting value, with the width of the confidence interval also being obtained for larger k (assuming $n \geq k$), and calculating n by $\frac{N}{k}$. The width of the confidence interval is obtained using simulations for each combination of n and k, utilizing any of the confidence interval method mentioned in Section 3. The combination which provides the minimum width of the confidence interval for ρ is chosen.
3. The width of the confidence interval obtained for a specific n and k might not be sufficiently small. If this is the case, larger values of N are used and Step 2. is repeated. In case the width is much less than ω, smaller values of N are used.
4. Finally, from the above sets of N, the minimum N, and the associated optimal combination of n and k that satisfies the condition that the width of the confidence interval is less than ω is selected.

References

1. Fleiss, J. & Shrout, P. Approximate interval estimation for a certain intraclass correlation coefficient. *Psychometrika*. **43**, 259-262 (1978), https://doi.org/10.1007/BF02293867

2. McGraw, K. & Wong, S. Forming inferences about some intraclass correlation coefficients. *Psychological Methods*. **1**, 30-46 (1996)

3. Satterthwaite, F. An Approximate Distribution of Estimates of Variance Components. *Biometrics Bulletin*. **2**, 110-114 (1946), http://www.jstor.org/stable/3002019

4. Zou, K. & McDermott, M. Higher-moment approaches to approximate interval estimation for a certain intraclass correlation coefficient. *Statistics In Medicine*. **18**, 2051-2061 (1999)

5. Xiao, Y. & Liu, H. Modified profile likelihood approach for certain intraclass correlation coefficients. *Computational Statistics*. **28**, 2241-2265 (2013), https://doi.org/10.1007/s00180-013-0405-x

6. Saito, Y., Sozu, T., Hamada, C. & Yoshimura, I. Effective number of subjects and number of raters for inter-rater reliability studies. *Statistics In Medicine*. **25**, 1547-1560 (2006)
